# A gas-enshrouded and gas-reddened black hole at cosmic dawn

**DOI:** 10.1038/s41586-026-10846-4

**Published:** 2026-08-12

**Authors:** Rohan P. Naidu, Jorryt Matthee, Harley Katz, Anna de Graaff, Pascal A. Oesch, Aaron Smith, Jenny E. Greene, Gabriel Brammer, Andrea Weibel, Raphael Hviding, John Chisholm, Ivo Labbé, Robert A. Simcoe, Callum Witten, Wendy Q. Sun, Hakim Atek, Josephine F. W. Baggen, Sirio Belli, Rachel Bezanson, Leindert A. Boogaard, Sownak Bose, Rychard J. Bouwens, Alba Covelo-Paz, Pratika Dayal, Yoshinobu Fudamoto, Lukas J. Furtak, Emma Giovinazzo, Andy Goulding, Max Gronke, Kasper E. Heintz, Michaela Hirschmann, Garth Illingworth, Akio K. Inoue, Benjamin D. Johnson, Joel Leja, Ecaterina Leonova, Ian McConachie, Michael V. Maseda, Priyamvada Natarajan, Erica Nelson, David J. Setton, Irene Shivaei, David Sobral, Mauro Stefanon, Sandro Tacchella, Sune Toft, Alberto Torralba, Pieter van Dokkum, Arjen van der Wel, Marta Volonteri, Fabian Walter, Bingjie Wang, Darach Watson, Katherine Whitaker

**Affiliations:** 1 MIT Kavli Institute for Astrophysics and Space Research, Cambridge, MA USA; 2 Institute of Science and Technology Austria (ISTA), Klosterneuburg, Austria; 3 Department of Astronomy and Astrophysics, University of Chicago, Chicago, IL USA; 4 Max-Planck-Institut für Astronomie, Heidelberg, Germany; 5 Department of Astronomy, University of Geneva, Geneva, Switzerland; 6 Cosmic Dawn Center (DAWN), Copenhagen, Denmark; 7 Niels Bohr Institute, University of Copenhagen, Copenhagen, Denmark; 8 Department of Physics, The University of Texas at Dallas, Richardson, TX USA; 9 Department of Astrophysical Sciences, Princeton University, Princeton, NJ USA; 10 Department of Astronomy, The University of Texas at Austin, Austin, TX USA; 11 Centre for Astrophysics and Supercomputing, Swinburne University of Technology, Melbourne, Victoria Australia; 12 Institut d’Astrophysique de Paris, CNRS, Sorbonne Université, Paris, France; 13 Department of Astronomy, Yale University, New Haven, CT USA; 14 Dipartimento di Fisica e Astronomia, Università di Bologna, Bologna, Italy; 15 Department of Physics and Astronomy and PITT PACC, University of Pittsburgh, Pittsburgh, PA USA; 16 Leiden Observatory, Leiden University, Leiden, The Netherlands; 17 Institute for Computational Cosmology, Department of Physics, Durham University, Durham, UK; 18 Kapteyn Astronomical Institute, University of Groningen, Groningen, The Netherlands; 19 Center for Frontier Science, Chiba University, Chiba, Japan; 20 Department of Physics, Ben-Gurion University of the Negev, Be’er-Sheva, Israel; 21 Max Planck Institute for Astrophysics, Garching, Germany; 22 Institute of Physics, Lab for galaxy evolution and spectral modelling, EPFL, Observatory of Sauverny, Geneva, Switzerland; 23 Department of Astronomy and Astrophysics, University of California Santa Cruz, Santa Cruz, CA USA; 24 Department of Physics, School of Advanced Science and Engineering, Faculty of Science and Engineering, Waseda University, Tokyo, Japan; 25 Waseda Research Institute for Science and Engineering, Faculty of Science and Engineering, Waseda University, Tokyo, Japan; 26 Center for Astrophysics — Harvard and Smithsonian, Cambridge, MA USA; 27 Department of Astronomy and Astrophysics, The Pennsylvania State University, University Park, PA USA; 28 Institute for Computational and Data Sciences, The Pennsylvania State University, University Park, PA USA; 29 Institute for Gravitation and the Cosmos, The Pennsylvania State University, University Park, PA USA; 30 GRAPPA, Anton Pannekoek Institute for Astronomy and Institute of High-Energy Physics, University of Amsterdam, Amsterdam, The Netherlands; 31 Department of Astronomy, University of Wisconsin-Madison, Madison, WI USA; 32 Department of Physics, Yale University, New Haven, CT USA; 33 Yale Center for Astronomy and Astrophysics, Yale University, New Haven, CT USA; 34 Department for Astrophysical and Planetary Science, University of Colorado, Boulder, CO USA; 35 Centro de Astrobiología (CAB), CSIC-INTA, Madrid, Spain; 36 BNP Paribas Corporate & Institutional Banking, Lisbon, Portugal; 37 Departamento de Física, Faculdade de Ciencias, Universidade de Lisboa, Lisbon, Portugal; 38 Departament d’Astronomia i Astrofísica, Universitat de València, València, Spain; 39 Unidad Asociada CSIC ‘Grupo de Astrofísica Extragaláctica y Cosmología’ (Instituto de Física de Cantabria - Universitat de València), València, Spain; 40 Kavli Institute for Cosmology, University of Cambridge, Cambridge, UK; 41 Cavendish Laboratory, University of Cambridge, Cambridge, UK; 42 Sterrenkundig Observatorium, Universiteit Gent, Ghent, Belgium; 43 Department of Astronomy, University of Massachusetts, Amherst, MA USA; 44 Present Address: Institute for Astronomy, University of Hawai‘i, Honolulu, HI USA

**Keywords:** Early universe, Galaxies and clusters

## Abstract

The physical processes that led to the formation of billion-solar-mass black holes within the first 700 million years of cosmic time, a period known as cosmic dawn, remain a puzzle^1^. Several theoretical scenarios have been proposed to seed and rapidly grow black holes^2–4^, but direct observations of these mechanisms remain elusive. Here we present a source 660 million years after the Big Bang that exhibits singular properties: among the largest hydrogen Balmer breaks reported at any redshift, broad multi-peaked Hβ emission, and Balmer line absorption in several transitions. We model this source as an enshrouded black hole in which the Balmer break and absorption features are a result of extremely dense, turbulent gas forming a dust-free envelope around a supermassive black hole^5,6^. This source may provide evidence of an early black hole embedded in dense gas—a theoretical configuration proposed to rapidly grow black holes by super-Eddington accretion^7,8^. Radiation from the black hole seems to dominate almost all observed light, leaving limited room for contribution from its host galaxy. If the source merged with its brighter neighbour, it would resemble the recently discovered ‘little red dots’ with perplexing spectral energy distributions^9–11^. The redness of the black hole is due to gas, not dust^12,13^, and scattering, not kinematics, gives rise to the complex line shapes and luminosities—black hole masses of these sources may therefore be overestimated by orders of magnitude.

## Abstract (web summary)

Observations of an unusual cosmic-dawn source illuminate how early supermassive black holes may have grown rapidly in a gas-enshrouded state; such enshrouded black holes may lie at the heart of the 'little red dots'.

## Main

**Fig. 1:**
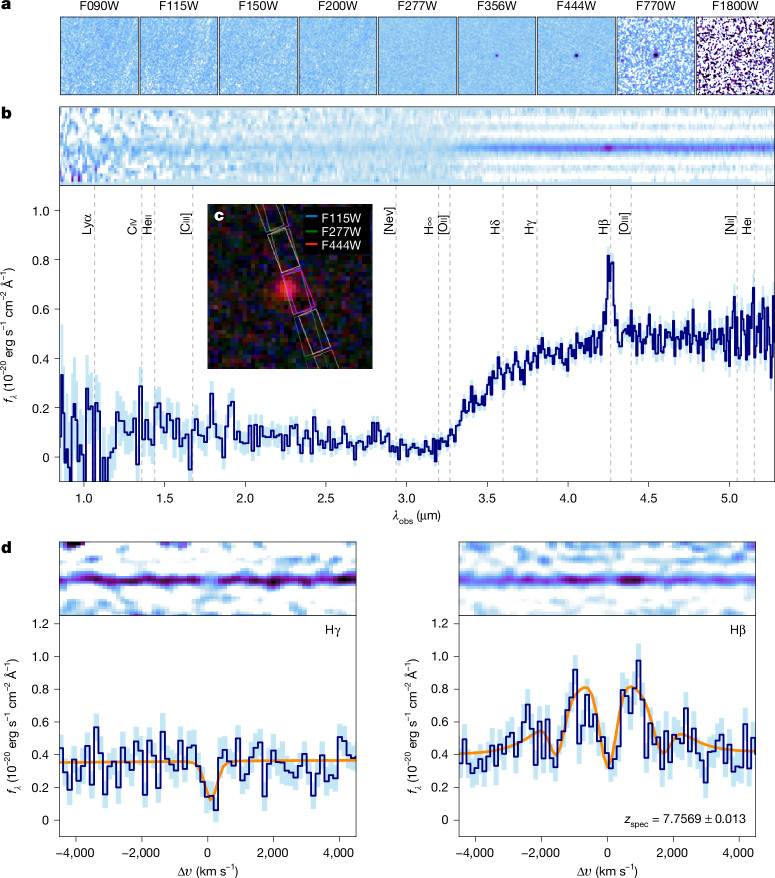
JWST imaging and spectroscopy of MoM-BH*-1. **a**, The 3 × 3″ NIRCam and MIRI images of MoM-BH*-1 spanning 0.9–18 μm. The source is point-like and detected (>3*σ*) only in the F356W, F444W and F770W bands, apparently disappearing in the bluer bands. **b**, The NIRSpec prism spectrum (dark blue) shows that the disappearance is due to an enormous Balmer break. Key spectral features such as the Balmer series are marked with dashed lines. **c**, The 1″ RGB image shows the almost identical slit positions with which the source was observed with the prism (**b**) and G395M grating (**d**). **d**, Deep absorption features in Hγ and Hβ are evident in the G395M grating spectra. The location of the central absorption is consistent across both Hβ and Hγ as well as across the prism and grating spectra. The systemic redshift is based on the [Oiii] 4,960, 5,008 Å doublet. A representative draw from the emission line model posterior is plotted in orange (Methods).

We recently observed MoM-BH*-1 with the NIRSpec instrument of JWSTas part of the ‘Mirage or Miracle’ (MoM) JWST program (GO-5224). MoM-BH*-1 was selected as a high-priority target for spectroscopic follow-up based on its striking appearance in NIRCam images of the Ultra Deep Survey (UDS) extragalactic field^14^. It stood out as the reddest source in this approximately 250 arcmin^2^ field (F277W-F356W > 2.5 mag), appearing remarkably luminous (F444W = 25.4 mag) and unresolved at >3 μm, while apparently disappearing at shorter wavelengths (F200W > 28.5 at 3*σ*; Fig. 1a).

Figure 1b shows the 4.5-h deep NIRSpec prism spectrum obtained (*R* ≈ 150, about 1−5 μm; 15 December 2024). Figure 1d shows a public archival 1.5-h NIRSpec G395M spectrum taken by the EXCELS survey^15^ (*R* ≈ 1,500, about 3−5 μm; 19 December 2023). The redshift (${z}_{{\rm{spec}}}=7.756{9}_{-0.0012}^{+0.0013}$) is confirmed by several features: a broad Hβ emission line ($\mathrm{FWHM}=3,03{6}_{-506}^{+361}\,\mathrm{km}\,{{\rm{s}}}^{-1}$), Hγ absorption at the same redshift as deep Hβ absorption and a strong Balmer break between 3 μm and 4 μm, where the flux drops by a factor of >20×, thereby explaining the extremely red NIRCam colour (${f}_{\mathrm{F444W}}^{\nu }/{f}_{\mathrm{F277W}}^{\nu } > 20$). A narrow [Oiii]4,960, 5,008 Å doublet is detected (3.5*σ*) at a redshift consistent with the Balmer lines.

**Fig. 2:**
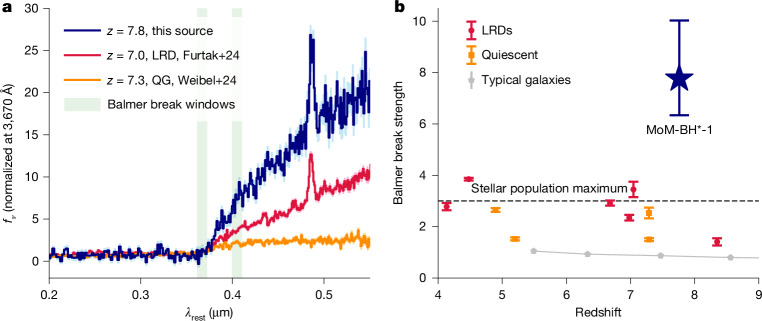
The exceptional Balmer break strength of MoM-BH*-1. **a**, Contrast of MoM-BH*-1 against a quiescent galaxy and an LRD that lie at a similar redshift (*z* ≈ 7) and exhibit some of the strongest Balmer breaks reported yet (about 3). The two wavelength windows we use to compute break strengths are highlighted in green—these windows ([3,620–3,720] Å and [4,000–4,100] Å) are free of strong emission lines and are particularly suited for studying high-redshift galaxies. The spectra shown here are normalized in the blue window—flux in this window is detected at >4.5*σ* for MoM-BH*-1. **b**, Comparison of break strengths of quiescent galaxies, LRDs with Balmer breaks and stacks of star-forming galaxies at similar redshifts as MoM-BH*-1. The dashed line represents the maximum break strength expected for a dust-free stellar population and a Chabrier initial mass function. MoM-BH*-1 displays the strongest Balmer break at these redshifts and lies well beyond this stellar population maximum. The asymmetric and higher uncertainty on the break strength in MoM-BH*-1 is due to the relatively fainter flux and lower signal-to-noise ratio (about 3) in the blue window.

The strength of the Balmer break is remarkable. In Fig. 2, we compare MoM-BH*-1 with objects exhibiting Balmer breaks at similar redshifts: quiescent galaxies and a compilation of ‘little red dots’ (LRDs; compact, red objects with broad Balmer lines). The maximum break strength expected for a dust-free stellar population assuming a typical initial mass function^16^ is 3 (refs. ^17,18^). As an extreme case, a population comprised purely of A-type stars with the strongest breaks would have a strength <5 (refs. ^19,20^). Crucially, MoM-BH*-1 is the only source that lies firmly beyond these limits with a break strength of $7.{7}_{-1.4}^{+2.3}$. A range of puzzling objects with a combination of broad Balmer lines and Balmer breaks have been discovered with JWST^13,17,21,22^. However, all these sources fall around or below the observed and theoretical maxima for stellar populations, thereby permitting a wide variety of interpretations ranging from pure stars to pure AGN, combinations thereof, as well as other explanations^6,17,23–25^. By contrast, it seems a relatively inescapable conclusion that the spectrum of MoM-BH*-1 does not arise from a stellar population.

The key to unravelling the break is the intense absorption in the Balmer lines (Hβ and Hγ), which occurs simultaneously with Hβ emission. Absorption in these non-resonant emission lines implies extreme gas densities (*n*_H_ ≳ 10^9^ cm^−3^) such that hydrogen atoms with populated *n* = 2 shells are abundant^5,26^. Before JWST, this phenomenon was observed only in a handful of sources hosting supermassive black holes (BHs)^26–28^, but it is now witnessed frequently in LRDs^9,11,21^. The absorption in MoM-BH*-1 is qualitatively similar to these LRDs, but is particularly strong, with missing flux at the line-centre and over a broad velocity range.

The point-source morphology (<100 parsecs in F356W, 95% upper limit) and extremely broad Hβ emission (comparable to luminous *z* > 6 quasars^29^) further imply a supermassive BH may be powering this source. Tentative detections of narrow forbidden lines (the [Oiii] doublet) and a hint of variability ($3{0}_{-7}^{+7} \%$ brightening in 56 days at 3−5 μm, albeit measured with different instruments; see Extended Data Fig. 5) add further evidence for a BH. Such an extreme Hβ/[Oiii]5,008 Å ratio >10 ($11.{4}_{-2.5}^{+4.2}$), consistent with high gas densities, in which [Oiii] is suppressed by collisional de-excitation (≳10^6^ cm^−3^), has been reported only in a single source at *z* > 6, which appears to be a broad-line, variable AGN^6,13,30^. However, no known object, AGN or not, displays the singular Balmer break we report in this source.

Motivated by the evidence for a supermassive BH and the strong Balmer absorption, following refs. ^5,6^, we construct a grid of Cloudy^31^ spectral synthesis models, in which we embed a classical AGN accretion disk^32^ within extremely dense gas (see the Methods for details). Informed by the width of the Hγ and Hβ absorption (full width at half maximum (FWHM) ≈ 300−500 km s^−1^), we model the absorbing gas with turbulent velocity.

**Fig. 3:**
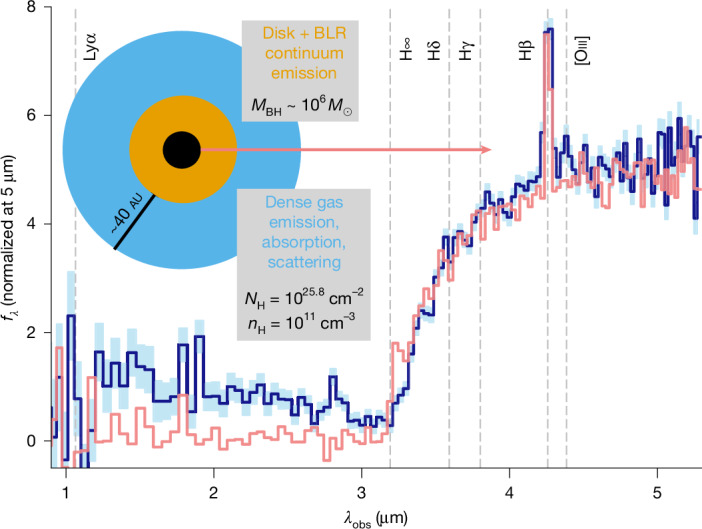
Comparison with a mock spectrum of a gas-enshrouded BH model. Schematic of an SMBH with a 40 au column of dense gas—the continuum is produced in hot regions close to the SMBH, whereas absorption, scattering and further emission occur in the dense gas atmosphere. The data (dark blue) are binned (3×) to emphasize that the continuum shape that our fiducial model (pink, with noise as per error spectrum) provides is an excellent match. The model is selected to reproduce the Balmer break strength and Balmer line EWs, while also matching the UV-faintness and MIRI long-wavelength data without having to invoke different mechanisms for lines and continuum. The narrow [Oiii] emission and additional UV luminosity plausibly arise from the faint host galaxy and are not captured by the model (Fig. 4). The excess flux around H_*∞*_ is a Cloudy model artefact because of modelling with a finite number of hydrogen levels.

With this simple, idealized model, we are able to match the key features in this source, including a deep, smooth Balmer break. Figure 3 shows our fiducial model selected from a grid of close to a million models and spanning the extreme parameters demanded by this source. The model is selected to reproduce the equivalent widths (EWs) of Hβ and Hγ, the Balmer break strength, the ultraviolet (UV) weakness (*M*_UV_ > −18.5), and the shape of the continuum out to *λ*_obs_ ≈ 20 μm constrained by Mid-Infrared Instrument (MIRI). Extremely dense gas (*n*_H_ = 10^11^ cm^−3^, *N*_H_ = 10^25.8^ cm^−2^) with a high turbulent velocity (500 km s^−1^) is necessary to produce these features. This velocity happens to match the width of the central absorption in Hβ. An important facet of this model compared with previous efforts^6^ is that negligible dust attenuation (*A*_V_ = 0.15 mag compared with, for example, *A*_V_ > 2 mag) is invoked to match the continuum shape, including the MIRI detection. This feature is consistent with stringent infrared constraints ruling out a dominant role for dust in the LRDs^33–35^. We emphasize that this modelling exercise is highly simplistic (for example, the intrinsic active galactic nuclei (AGN) spectral energy distribution (SED) may be vastly different than assumed^36^ or the structure may be convective^8^) and only serves to provide broad physical intuition that dense gas enveloping a central engine may account for the singular observed features.

The detailed structure of the emission lines holds important clues to the physical picture as well. In particular, the Hβ line profile is symmetric with peaks and troughs mirrored on either side of the systemic redshift (Fig. 1 and Extended Data Fig. 2). This makes it unlikely that random absorbers along the line of sight or inflows and/or outflows are responsible for the line structure. Instead, a coherent, symmetric absorption structure (such as a shell of gas) close to the object (as the central absorber is at the systemic velocity $4{2}_{-200}^{+80}\,\mathrm{km}\,{{\rm{s}}}^{-1}$) is our preferred solution for these features. In the Methods, we present a simple speculative model for this symmetry as arising from multiple scatterings of Hβ that behaves like Lyα.

In a much more extreme avatar of the Balmer breaks observed in galaxies due to absorption in stellar atmospheres^37^, here we have a BH seen through a dense, turbulent envelope of Compton thick gas spanning about 10−100 au. Although similarly dense gas is observed in broad-line regions, the remarkable enveloping configuration and the resulting SED is new. The SED has the characteristic features of an SMBH such as broad lines, but also features that are traditionally associated with stars such as a Balmer break, a blackbody-like SED and increasingly deep absorption across the Balmer series.

To confront the puzzle of approximately 10^9^*M*_⊙_ SMBHs that are already in place by *z* ≳ 7.5 (ref. ^38^), theories of SMBH growth have predicted channels of seeding massive BHs as well as growing them at a rapid, super-Eddington pace. As an early, growing BH (*M*_BH_ ≈ 10^6−7^*M*_⊙_; Methods), MoM-BH*-1 exhibits key features predicted by these models. For example, a class of models^7,8^ predicts that if a BH is ensconced in dense gas (for example, in a dense gas-rich nuclear star cluster or in a quasi-spherical gas distribution), the high opacity can trap the accretion radiation or ‘convect’ it away such that gravity may overcome radiative feedback and exceed the Eddington limit. MoM-BH*-1 may be undergoing an active super-Eddington burst, or perhaps it is in the end stages of such an episode in which the gas envelope that has nourished it is still in place. Naively applying local scaling relations^39^ under standard assumptions to this source indicates the latter scenario ($L/{L}_{{\rm{Edd.}}}=0.1{8}_{-0.03}^{+0.07}$), but accounting for the extreme conditions may favour the former (*L*/*L*_Edd_ ≈ 5 − 10; Methods).

**Fig. 4:**
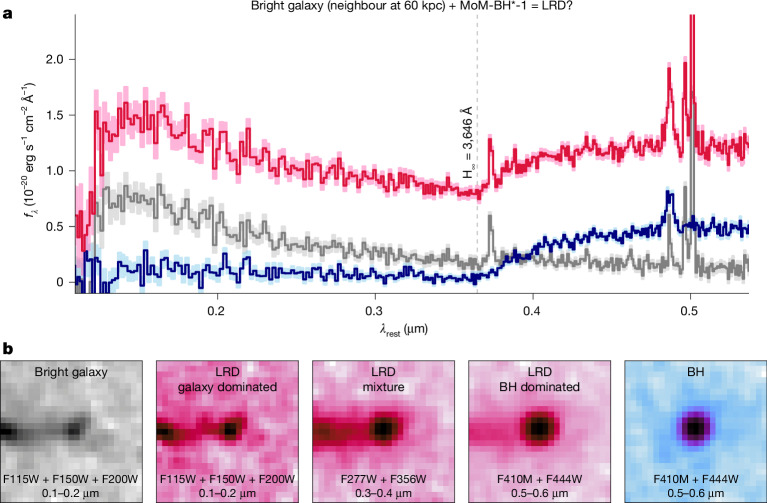
Explanation of LRDs as gas-enshrouded BHs embedded in comparably bright host galaxies. **a**,**b**, MoM-BH*-1 (blue) lies close to a *M*_⋆_ ≈ 10^9.5^ *M*_⊙_ galaxy at the same redshift (grey). These sources are expected to merge in about 100 Myr, and their superimposed spectrum (offset for clarity; **a**) and photometry (1″ × 1″ NIRCam stamps; **b**) is shown in red. The combined spectrum bears a striking resemblance to the typical LRD, displaying a V-shaped SED, an inflection around H_*∞*_ = 3,646 Å and a complex Hβ profile with a broad component. The NIRCam stamps demonstrate compactness in the rest-optical and an extended structure in the rest-UV. Although the galaxy is dominant in the rest-UV, the BH outshines it towards the rest-optical.

It is separately noteworthy that the faint host galaxy surrounding the BH is a low-mass (and hence, perhaps, metal-poor) dwarf galaxy (M_*_ < 10^8.5^*M*_⊙_). We derive this limit based on the observed UV luminosity that we scale empirically using a large spectroscopic reference sample of dwarf galaxies^40^. The UV flux is probably some mixture of BH and galaxy; hence this is an upper limit—but this is the wavelength at which the galaxy shines the brightest relative to the BH^41^. This low-mass galaxy appears to be associated to a more massive (about 10^9.5^*M*_⊙_) spectroscopically confirmed galaxy at a projected distance of only around 60 proper kpc and Δ*z* < 0.01 (Fig. 4). Theories of direct collapse BHs that form out of primordial gas predict that this proximity to an ionizing source may be the key to suppressing the formation of molecular hydrogen thereby aiding direct collapse^42^—if more BHs are found in similar configurations, this may be a telling sign. For now, we note that the formation of these enshrouded BHs remains an open question.

Overlaying the spectra and photometry of the BH and its nearby neighbour—expected to merge in about 100 Myr (ref. ^43^)—produces a V-shaped SED that corresponds to a point source in the rest-optical and an extended source in the rest-UV (Fig. 4). These are the defining characteristics of the numerous LRDs recently revealed by JWST^9^. The combined data shown in Fig. 4 satisfy the standard criteria used to define these sources^44^ and also show subtle features such as a Balmer break and inflection around H*∞*^45^ along with a broad Balmer line with absorption^9^.

Explaining the unique constellation of features found in LRDs has been challenging, with no known observed combination of star-forming galaxies and AGN accounting for all these properties self-consistently^21,24^. Based on the exercise in Fig. 4, we propose that treating MoM-BH*-1 as an effectively pure template for the AGN component of LRDs and combining it with a star-forming galaxy of matched UV-brightness accounts for the puzzling LRD properties. In this composite picture, the star-forming galaxy dominates in the UV (extended morphology and narrow Lyα line), whereas the BH dominates in the rest-optical (Balmer break, broad Balmer lines and variability expected from AGN around Hα). X-ray weakness may be understood as a consequence of the Compton thick gas envelope while the FIR-weakness is a consequence of much lower dust attenuation than all LRD studies assume because the BH SED is intrinsically UV-weak below the Balmer break. The diversity in the relative contribution and properties of each component (for example, star-formation history of the host, gas density around the BH) may account for the full diversity seen in LRD properties. A previous study^46^ shows electron scattering in dense gas may be common across LRDs—this is independent evidence that gas-enshrouded BHs like the source studied here may be the central engines of these enigmatic sources.

## Methods

### Observations and data reduction

MoM-BH*-1 has been observed with JWST by three programmes. It was imaged in cycle 1 by the PRIMER survey^14^ (JWST-GO-1837; principal investigator: J. Dunlop) using the MIRI (5 January 2023 and 16 January 2023) and NIRCam (7 August 2023 and 9 August 2023) instruments. In cycle 2 (19 December 2023), the EXCELS survey^15^ (JWST-GO-3543) obtained 1.5 h of NIRSpec G395M spectroscopy. In cycle 3 (15 December 2024), we targeted MoM-BH*-1 as part of the ‘Mirage or Miracle’ NIRSpec prism survey (JWST-GO-5224). We included MoM-BH*-1 as a high-priority target second in importance only to luminous *z* > 10 sources in our UDS masks, because it appeared in several priority target lists—AGN/LRD candidates selected based on compact morphology and template fitting with EAZY^47^, extremely massive galaxy candidates, sources with peculiar red colours and the literature LRD candidates^48^. 

We use the v.7.2 images of the PRIMER field released on the DAWN JWST archive (DJA) reduced using the grizli software^49^. PSF-matched photometric catalogues based on these images were produced in ref. ^50^. We use the public v.3 NIRSpec reductions of the EXCELS grating data from the DJA derived using the msaexp software^37,51,52^. The MoM data are reduced with the same pipeline following the same choices.

Although we found no relevant radio or ALMA archival data, MoM-BH*-1 has been observed with Chandra^53^. Similar to virtually all LRDs^9,54,55^, it remains undetected in the X-rays (*L*_*x*_ < 44.5 erg s^−1^ (1*σ*) at rest frame 5–90 keV). Key empirical properties of the source are summarized in Extended Data Table 1.

### Emission line fitting

We use a custom NIRSpec emission line fitting package^56^ to simultaneously fit emission lines in the grating and prism spectra. The advantage of this approach is that despite the low signal-to-noise ratio (SNR) in either mode, features may be robustly recovered because of their occurrence at the same wavelength across both dispersers.

**Extended Data Fig. 1:**
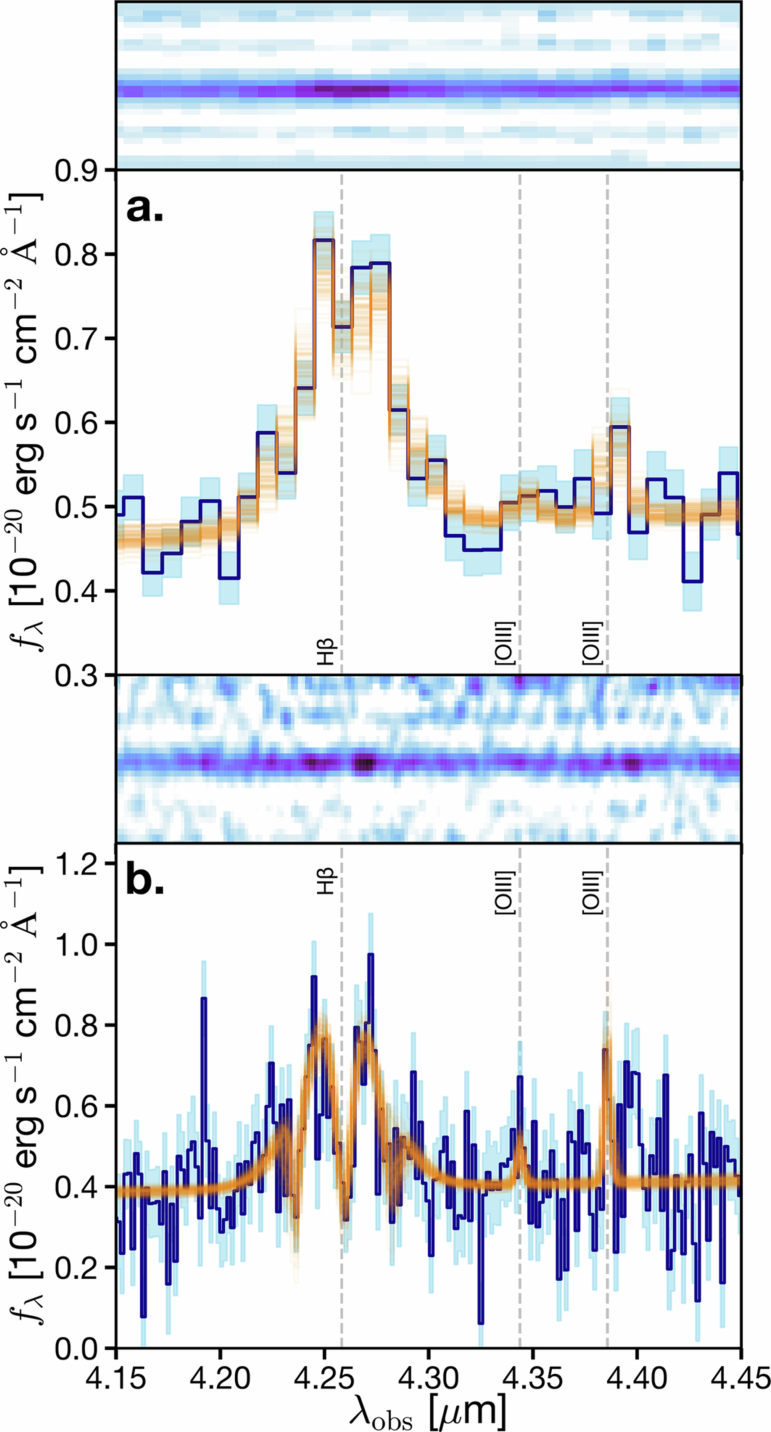
Simultaneous emission line fits to the prism (Panel a.) and grating (Panel b.) spectra. 100 draws from the posterior are shown in orange. The consistency of features across both modes inspires confidence in their reality. For example, the detailed structure of the H*β* line – a central absorber, extremely broad wings, and absorption even in the wings – recurs in both panels. Similarly, the existence of narrow [Oiii] 5008 Å emission would be difficult to discern in either mode by itself, but is recovered in the joint fit.

**Extended Data Fig. 2:**
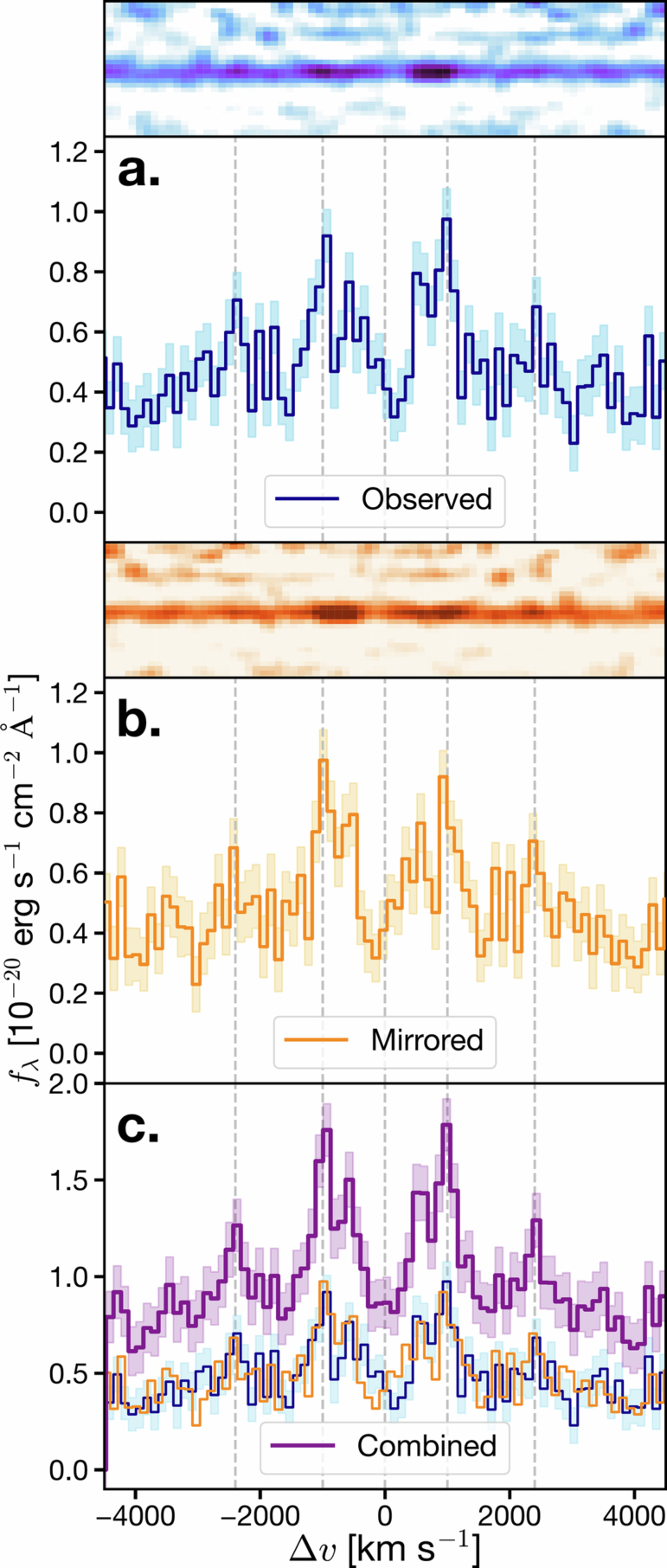
The remarkable symmetry of the H*β* line profile points to a symmetric configuration of absorbing gas. Here we compare the observed line profile (Panel a, blue) to a line profile mirrored around the systemic redshift derived from [Oiii] (Panel b, orange). In Panel c we co-add these spectra. These profiles display peaks (± 1000 km s^−1^, and perhaps also ± 2400 km s^−1^) and troughs (± 1500 km s^−1^; see absorber locations in Extended Data Table 2) at similar velocities. This potential symmetry has important implications. First, it means the recovered systemic redshift from [Oiii] is robust. Furthermore, these features are highly unlikely to be the result of random absorption systems or inflows/outflows that are fortuitously aligned at the same positive and negative velocities. Instead, this alignment suggests the presence of a symmetric absorbing structure (e.g., shells of gas).

We first fit the Hβ line and [Oiii] doublet, and then use the redshift as a prior to fit Hγ (Fig. 1). We model Hβ as a single emission line with three absorbers constrained to have negative flux (one at line-centre and two on either side of zero velocity) motivated by the symmetric absorption troughs on either side of the central double-peak (Extended Data Fig. 2). The systemic redshift is tied to [Oiii] and the broad Hβ component, with the absorbers allowed to range freely. The number of absorbers is decided based on the maxima reached in the reduced *χ*^2^, which is similar for three and four absorbers, but we opt for parsimony. This large number of absorbers may be merited to account for secondary peaks at ±2,400 km s^−1^ (Extended Data Fig. 2). Including narrow Hβ at the systemic redshift leads to completely unconstrained flux degenerate with absorption and no improvement, so we neglect this component. Resulting fits, in which we sample the posterior with the NUTS sampler implemented in numpyro^57^, are shown in Extended Data Fig. 1 and reported in Extended Data Table 2.

Although the formal errors on the derived fluxes and line widths of many of the components is significant, the key features relevant to this analysis are robustly recovered—extremely broad Hβ emission, and deep, broad absorption wiping out about 25% of the emission flux, including approximately 100% of the flux at line-centre. Another notable aspect of these fits is the location of the two absorbers—they are recovered at very similar velocities, but on either side of line-centre (approximately ±1,500 km s^−1^), albeit with significant uncertainties ($-1,53{2}_{-113}^{+345}\,\mathrm{km}\,{{\rm{s}}}^{-1}$ and $+1,55{6}_{-1,378}^{+232}\,\mathrm{km}\,{{\rm{s}}}^{-1}$). We explore this symmetry, which extends not only to the location of the absorbers but also to the detailed structure of the entire emission line profile over a few 1,000 km s^−1^ in Extended Data Fig. 2.

### Morphology

We use pysersic^58^ to fit a Sersic profile to the imaging. We focus on the F356W and F444W imaging, in which the source is well-detected. We follow the procedure described in ref. ^59^—we build an empirical PSF using stars in the field, and then use this PSF with pysersic to sample the posterior Sersic parameters with numpyro. The source is unresolved, and we are able to place a 99% upper limit on the effective radius of <117 pc consistent with the BH-dominated interpretation.

### Cloudy modelling

That extremely dense gas might blanket the LRDs was already inferred in the work that defined this class of sources^9^—about 10% of these LRDs showed clear signs of Balmer absorption, with this being a lower limit due to resolution and SNR^11^ as opposed to the ≪0.1% in pre-JWST AGN^60^, with only a handful examples^9^. Local AGN^61^ do not display Balmer breaks anywhere as strong as MoM-BH*-1 (about 8), with the strongest breaks (≲2.5) occurring in quiescent galaxy AGN that reach the stellar population maximum shown in Fig. 2b. Inspired by Balmer absorption in LRDs, and using Cloudy models for continuum absorption^5^, demonstrated that the very gas producing strong Balmer absorption likely also produces Balmer breaks. However, note the breaks in these models are weaker than what we require to explain MoM-BH*-1 and are abrupt instead of the smooth rollover seen in this source. Here we build on this work.

The grid of parameters we explore is summarized in Extended Data Table 3. This grid spans a wide range to capture the extreme spectral shape at hand. We start with an intrinsic AGN continuum SED that is parametrized using a series of power laws and with a ‘big bump’ temperature^32^. This AGN continuum is passed through gas surrounding the central source that is defined in terms of its gas density, column density, metallicity and turbulent velocity. The irradiation of the cloud is modulated by an ionization parameter (log(*U*)). A large turbulent velocity of the absorbing gas 500 km s^−1^ is motivated by the width of the absorption lines we observe (see Fig. 1). This implies a high Mach number, which is consistent with recent models of AGN disks^62^—whether such high turbulence can be sustained across a large envelope remains to be seen. The turbulence is important in producing a smooth rollover instead of a sharp break^6^. Finally, we apply a uniform dust screen^63^ with *A*_V_ = 0−3 in the post-processing—this is the only post-processing step we apply.

To select models that are consistent with the data, we require: (1) Hβ in net emission with 30 < EW [Å] < 45; (2) Hγ in absorption with −5 < EW [Å] < 0 Å; (3) a strong Balmer break and high optical-to-UV ratio such that ${f}_{{\rm{4.5\mu m}}}^{\lambda }/{f}_{{\rm{1.8\mu m}}}^{\lambda } > 6$ and ${f}_{{\rm{4.5\mu m}}}^{\lambda }/{f}_{{\rm{2.8\mu m}}}^{\lambda } > 3$; and (4) fluxes in MIRI bands within 2*σ* of the observations. The few thousand models that satisfy these constraints are re-simulated at higher resolution, retaining only hydrogen that is relevant to the key features for simplicity and speed. Of these, we select the one that closely follows the detailed shape of the continuum. Furthermore, we experiment with the covering factor and distance between the gas and central source, and find these quantities to be degenerate with the AGN SED and ionization parameter. We find the ‘net transmitted’ flux (that is, the sum of the attenuated incident continuum and diffuse continua or lines) produces a good match to the data, not the ‘total’ flux (which also includes reflected continua or lines).

### Key features of fiducial Cloudy model: blanketed by gas, not by dust

**Extended Data Fig. 3:**
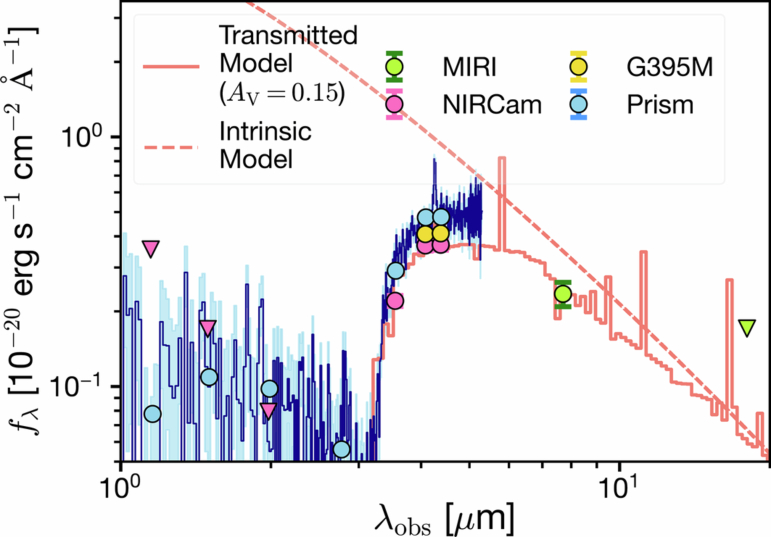
Comparison of fiducial model against long-wavelength data. Not only is this virtually dust-free model (*A*_V_ = 0.15 mag) able to explain the strong Balmer break and UV-weakness, but also the ‘turn-over’ at infrared wavelengths traced by MIRI (shown in green) and generically reported for the LRDs. The model spectrum (salmon) is normalized to the NIRCam fluxes that are closest to the epoch of the MIRI observations (see text and Extended Data Fig. 6 for discussion of variability). An important implication of this SED shape is that the bolometric luminosity is effectively entirely emitted in the rest-optical and near-IR.

First, we emphasize that this model (Fig. 3 and Extended Data Fig. 3) is a model that matches various features of interest based on our limited grid. The relevant parameter space that we have done our best to sample is high-dimensional, degenerate and much remains unknown (for example, the intrinsic SEDs of early AGN with effects such as photon trapping). Therefore, we caution against detailed inferences beyond the feasibility of the broad physical picture that we present here (an accretion disk embedded in dense gas).

Notably, the selected model features an extreme column density (about 10^25.8^ cm^−2^) comparable to the most enshrouded systems observed^64^ and a high gas density (10^11^ cm^−3^) conducive to Balmer absorption. The turbulent velocity of 500 km s^−1^ is commensurate with the width of the central Hβ absorber. Metal-poor gas expected of a dwarf galaxy at *z* ≈ 8 is preferred. The AGN slope parameters are within the range of literature SEDs^6,65^.

An important feature of the fiducial model is that it is virtually dust-free (*A*_V_ = 0.15 mag). The UV-weakness arises entirely because of the extreme hydrogen opacity. This is a crucial constraint on LRD models that typically invoke significant amounts of dust to suppress strong UV emission from classical AGN^21,24,66^ or to explain the weakness of Hβ relative to Hα^12^. Although significant *A*_V_ ≈2–3 helps dense gas AGN models produce a smooth Balmer break in the rest-optical^6^, this is ruled out by longer wavelength constraints in our source (Extended Data Fig. 3) and more generally by stringent infrared constraints on the LRDs^34,35^. We note that given the very low *A*_V_ ≈ 0, the exact details of the dust geometry (for example, screen compared with clumps) do not have a bearing on our results.

### Resonant Balmer scattering and insights from Lyα-like shell models

The Hβ profile of MoM-BH*-1 bears a remarkable resemblance to double-peaked Lyα^67–69^. This motivates us to explore whether Hβ is behaving like a resonant line under the high densities in MoM-BH*-1. It may be the case that this is a common phenomena in the LRDs, but that a symmetric double-peaked line with large separation is easier to observe in MoM-BH*-1 because of negligible Hβ emission from the sub-dominant host galaxy.

Although Hβ normally has an easy cascade escape route, saturating the 2*p* state can effectively trap Hβ photons, which may be plausible through Lyα pumping in these optically thick environments^70^. We test this idea with the COLT^71^ radiative transfer code with which we implement a simple shell model (directly analogous to Lyα shell models) for Hβ. The dominant parameters in these calculations are the thermal velocities of the inner and outer shells, the relative velocities between the shells and the optical depth encountered by Hβ. Strong turbulence is not accounted for in these toy models.

**Extended Data Fig. 4:**
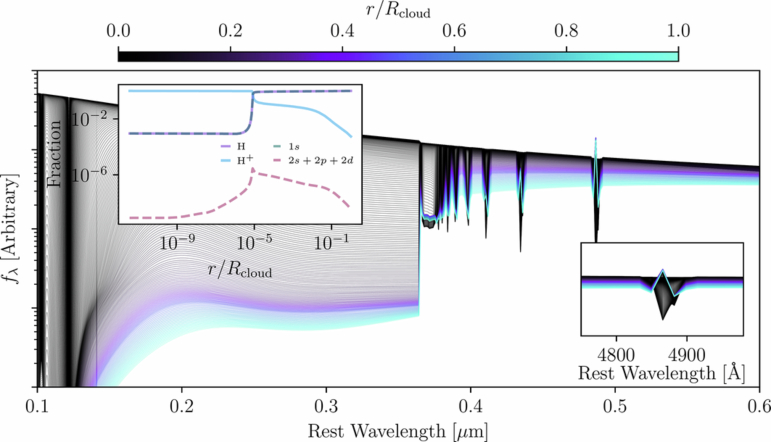
Cross-sectional view of the best-fit Cloudy model. Emergent spectra as observed at various depths are overlaid in the main panel – the incident power-law continuum (black, *r*/*R*_cloud_ ≈ 0) is reprocessed such that a deep Balmer break and absorption features are imparted (light blue, *r*/*R*_cloud_ ≈ 1). The inset panel in the top-left shows the fractional density of ionized gas (H^+^), neutral gas (H), and the *n* = 1 and *n* = 2 levels – crucially, a significant *n* = 2 population exists to produce deep Balmer absorption. Note that there is no dust in the model, and we apply dust only as a post-processing step. The bottom-right inset panel shows where the observed H*β* line arises from – remarkably, H*β* in emission is produced close to the surface (e.g., due to collisional effects) and has little to do with the kinematics of the interior – this has critical implications for BH mass estimates based on Balmer lines.

In Extended Data Fig. 4, we show how a relatively narrow Gaussian Hβ line manifests as a double-peaked profile. We confirm the basic trends seen with the Lyα line hold up for Balmer resonant scattering and can serve as a powerful guide to interpret MoM-BH*-1. With this simple toy model, we are able to generate a match for the velocity separation and intensity of the strongest peaks seen in the MoM-BH*-1 Hβ profile.

If scattering is underway, this has some important implications for the physics of the situation. The observed width of Balmer lines (especially when fitted as an absorption system to a broad Gaussian line; that is, the most common baseline model^9,21,72^) may not trace the kinematics of the broad-line region but are a consequence of resonant scattering. Therefore, SMBH masses in these systems that are based on line widths of the Balmer lines may be severely overestimated by up to 2 dex (Extended Data Table 4). More sophisticated treatments of resonant scattering effects in LRDs^73^ differ in assumptions and details, but make the same basic point.

### SMBH properties and ramifications for LRD parameters

Given the unique conditions (for example, unusual gas density) in this source, we must exercise care in applying the local scaling relations typically used to derive SMBH properties. Here we explore implications for a variety of approaches to back out the SMBH properties that are summarized in Extended Data Table 4. Given the significant systematic uncertainties, these calculations must be seen as order-of-magnitude estimates to bracket the range of possibilities.

First, we simply apply Hβ-based local scaling relations^39^ to the absorption-corrected and dust-corrected Hβ line. The absorption correction is typically done by emission line fitting assuming an underlying Gaussian or Lorentzian^9,21^ (as in Extended Data Fig. 1). For the dust correction, note that the Hα and Hβ line ratio^13^ is challenging to use given that their observed fluxes reflect radiative transfer in the dense gas. The standard approaches used in the literature at the moment infer a significant *A*_V_ of a few magnitudes either from continuum slope fitting or through SED modeling^6,10,12,13^. Going by the optical continuum slope^10^ would imply an *A*_V_ ≈ 2 for MoM-BH*-1 implying a BH mass of about 10^8.3^*M*_⊙_. This is comparable to the stellar mass of the host galaxy (<10^8.5^*M*_⊙_ at 95% confidence), that is, this is an apparently ‘overmassive’ BH relative to local scaling relations between host galaxy mass and BH mass^13,41^.

However, a key insight from the SED of MoM-BH*-1 is that the *A*_V_ required to explain the LRDs is likely negligible as the SED is intrinsically UV-weak below the Balmer break. That is, the LRDs are red not because of obscuration from dust, but because of opacity from gas. Accounting for this, with *A*_V_ = 0, the SMBH mass derived now is about 5 × 10^7^*M*_⊙_ with a luminosity of approximately 15% the Eddington limit.

**Extended Data Fig. 5:**
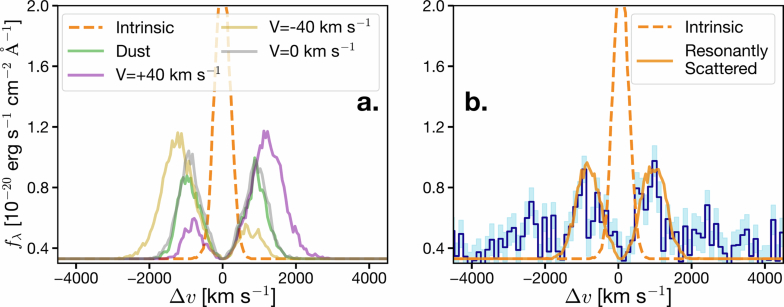
Resonant Balmer scattering?. **Panel a**. Through simple radiative transfer calculations with a shell model we demonstrate that H*β* behaves like Ly*α* if radiative decay transitions into the 2p state are suppressed. The trends that are well-known in the Ly*α* literature and relevant to our situation hold here. A narrow intrinsic line is scattered into double peaks. Dust weakens the blue peak preferentially, inflows/outflows boost one peak relative to another, and static shells produce equally strong peaks. **Panel b**. We model the two main peaks of the MoM-BH*-1 profile with a static, dust-free shell. This toy model does not capture the full extent of the broad wings which implies models with a more complicated geometry need to be explored, especially with higher S/N data.

Next, we note that the observed line width (after correcting for absorbers) may not be faithfully tracing the broad-line region, violating the basic ansatz for scaling relations (Extended Data Figs. 4 and 5). The underlying BH emission line profile in LRDs may be as complex as the one shown in Extended Data Fig. 2, but is perhaps difficult to disentangle from strong emission from the host. The complex structure may signify radiative processes that are modifying the emission arising from the broad-line region. As an example of one such process, if Hβ is undergoing resonant scattering through the atmosphere around the BH, then the intrinsic BLR width before scattering could be as low as around 600 km s^−1^ (Extended Data Fig. 5). This would yield an SMBH mass of approximately 10^6^*M*_⊙_.

Finally, we are able to constrain the BH properties using our Cloudy model. The bolometric luminosity directly follows from integrating the SED shown in Extended Data Fig. 3 (about 10^44.5^ erg s^−1^). We note that this is an order of magnitude lower than the value expected from local calibrations, further underscoring their inapplicability. We can convert this luminosity into a mass by observing that theoretical models of similar scenarios^74–76^ predict close to Eddington or super-Eddington accretion to sustain these convective envelopes. Furthermore, the ubiquity of OI emission thought to arise from Lyβ fluorescence in LRDs^21,66,77^ is thought to be a hallmark of super-Eddington accretion^78^. Inspired by these lines of evidence, requiring *L*/*L*_bol_ ≈ 1, therefore, allows us to estimate the BH mass as approximately 10^6.3^*M*_⊙_. Correcting the potentially overestimated literature BH masses by about 10−100× would bring the typical *M*_BH_/*M*_⋆_ of JWST AGN (about 1–10%; refs. ^79–81^) closer to the ratio in the local Universe (0.01%; ref. ^82^).

### Host galaxy properties

To constrain the host mass, we can leverage the insight from clustering analyses that the rest-UV light in the typical LRD (FWHM = 1,000−2,000 km s^−1^), on average, originates almost entirely from the host^41^. Of course, this is not true for all LRDs, particularly the most luminous sources with higher FWHM broad lines that display AGN signatures even in the rest-UV^21^, but so far they seem the exception. To construct an empirical *M*_⋆_−*M*_UV_ relation, we use the compilation of low-luminosity galaxies at *z* = 3−7 from the All the Little Things (ALT) survey^40^ in the Abell-2744 field. This is the largest spectroscopic sample of *M*_UV_ < −15 galaxies at these redshifts. The ALT stellar masses are derived with the Prospector SED fitting code applied to 27 bands of NIRCam + HST photometry, including all JWST medium and broad bands^83,84^. We estimate that a source with *M*_UV_ between −17.9 and −18.3 has a median stellar mass of $\log ({M}_{\star }/{M}_{\odot })=7.{4}_{-0.3}^{+0.6}$ and a 95% upper limit of $\log ({M}_{\star }/{M}_{\odot }) < 8.5$.

### Hints of variability

It is of particular interest to test for signs of variability in MoM-BH*-1 as an independent constraint on the physics of the source. In LRDs, the SED is a summation of the host and BH at all wavelengths to varying degrees, but in this case, the stark outshining of the host galaxy means an ‘undiluted’ variability signal may be stronger and more easily detected. One of the handful reports of variability in an LRD to date has been in a source with a large Balmer break implying a high BH fraction^30^.

**Extended Data Fig. 6:**
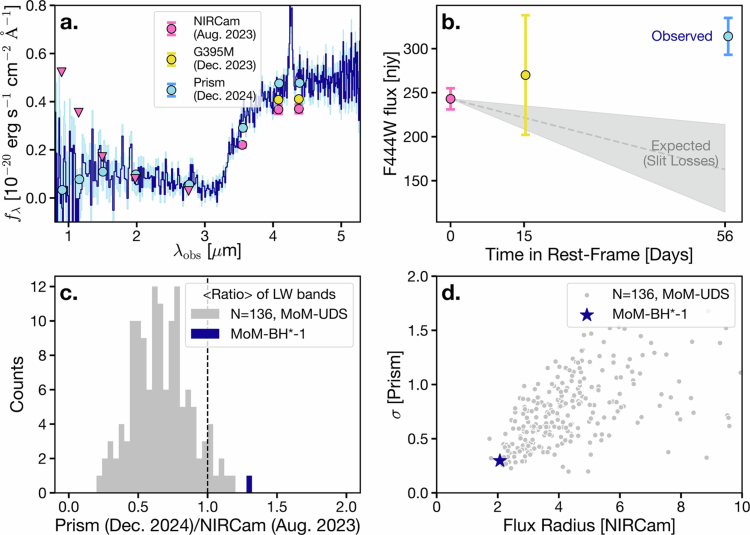
Testing variability in MoM-BH*-1. **Panel a**. Synthesized NIRCam photometry from the three epochs of observations are compared (pink, gold, blue) against the prism spectrum (navy). Non-detections in the NIRCam imaging (2*σ* upper limits) are shown as pink triangles. Note that the spectrum is generally deeper than the photometry, particularly in the rest-UV. **Panel b**. The observations span 56 days in the rest-frame of the source. While virtually every source in the prism observations has lost flux relative to the F444W imaging due to slit-losses (1*σ* contours for median ratio shown in grey), MoM-BH*-1 has brightened by  ≈ 30%. **Panel c**. MoM-BH*-1 is the only source across both observed masks in the UDS that has brightened by this degree relative to its NIRCam photometry. The ratio shown here is the median ratio of the flux synthesized from the prism and directly measured by NIRCam in the four LW filters observed by the PRIMER survey in August 2023 (F277W, F356W, F410M, and F444W). **Panel d**. This variability signal is unlikely to be due to an overestimated extraction aperture (*σ*) in the NIRSpec reduction pipeline. The compact flux radius of the source measured from the imaging is commensurate with the extraction aperture used for the spectra.

Three sets of observations covering 3−5 μm exist for MoM-BH*-1 separated by ≈ 60 days in the rest-frame (Extended Data Fig. 6). It is certainly not ideal to test for variability across three different observing modes with distinct systematics. Nevertheless, it is notable that the source appears to have brightened by 30 ± 7% between the first epoch (NIRCam) and third epoch (NIRSpec prism, no post-processing renormalization to photometry). The prism spectrum suffers from slit losses, and yet this source appears brighter than expected from the NIRCam photometry. It is the only source across the 136 sources with high-SNR photometry (SN > 10) and spectra (50^th^ percentile of SN > 5) observed in two masks as part of the MoM program that shows this degree of brightening. We also note that typically, the NIRSpec/G395M flux is systematically  ≈ 10–20% lower than the prism flux due to calibration uncertainties^85^ – accounting for this, both the prism and NIRSpec fluxes for MoM-BH*-1 appear to be in agreement and  ≈ 30% brighter than the NIRCam flux. The shape of the SED (and the depth of the Balmer break) is consistent across the NIRCam and prism data (for example, ${f}_{{\rm{F410M}}}^{\nu }/{f}_{{\rm{F356W}}}^{\nu }=2.0\pm 0.2$ in the prism vs. 2.2 ± 0.2 in NIRCam). The difference is in the absolute brightness.

Taken at face value, these measurements imply a ≈4*σ* detection of variability over a mere two months, marking MoM-BH*-1 as an excellent target for future monitoring campaigns. This may be independent evidence not only for the AGN nature of the source, but also for the volatile environment that exists around it.

## Online content

Any methods, additional references, Nature Portfolio reporting summaries, source data, extended data, supplementary information, acknowledgements, peer review information; details of author contributions and competing interests; and statements of data and code availability are available at 10.1038/s41586-026-10846-4.

## Supplementary information

Peer Review file

## Figures and Tables

**Extended Data Table 1:**
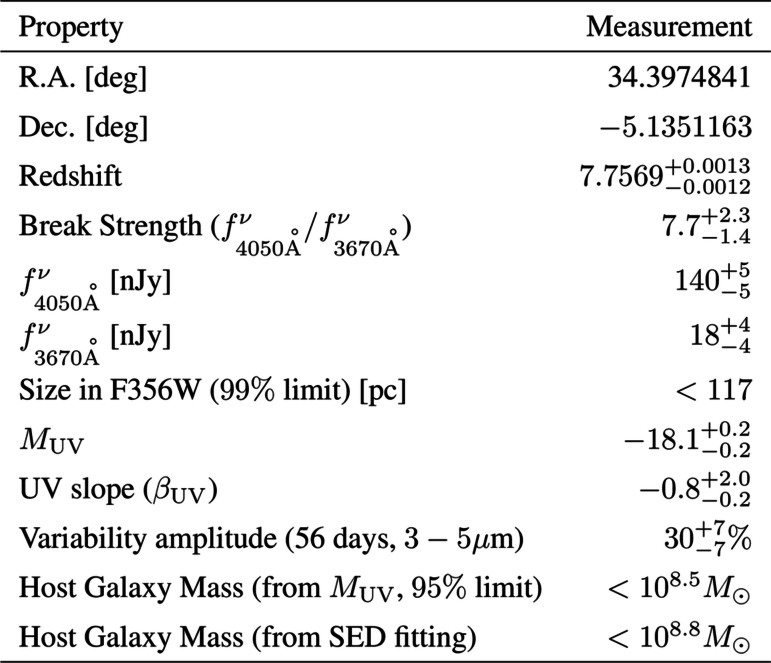
Summary of key properties

.

**Extended Data Table 2:**
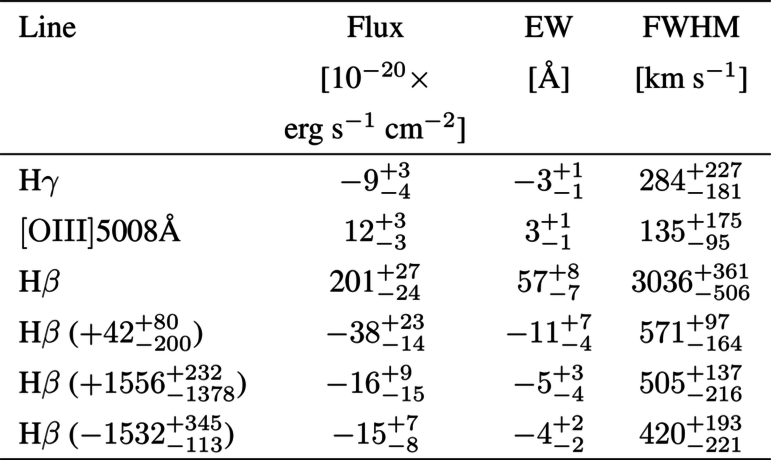
Emission and absorption line measurements

All lines are constrained to the systemic redshift tied to [Oiii], with the exception being the H*β* absorbers whose velocity offset in km s^−1^ is indicated in parentheses. Note the remarkably symmetric locations of the recovered H*β* absorption.

**Extended Data Table 3:**
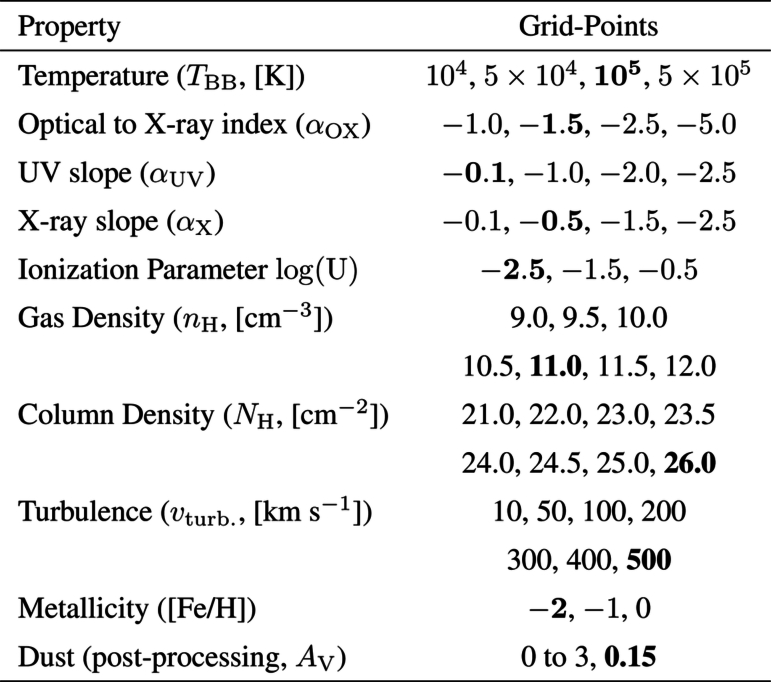
Grid of Cloudy parameters

**Extended Data Table 4:**
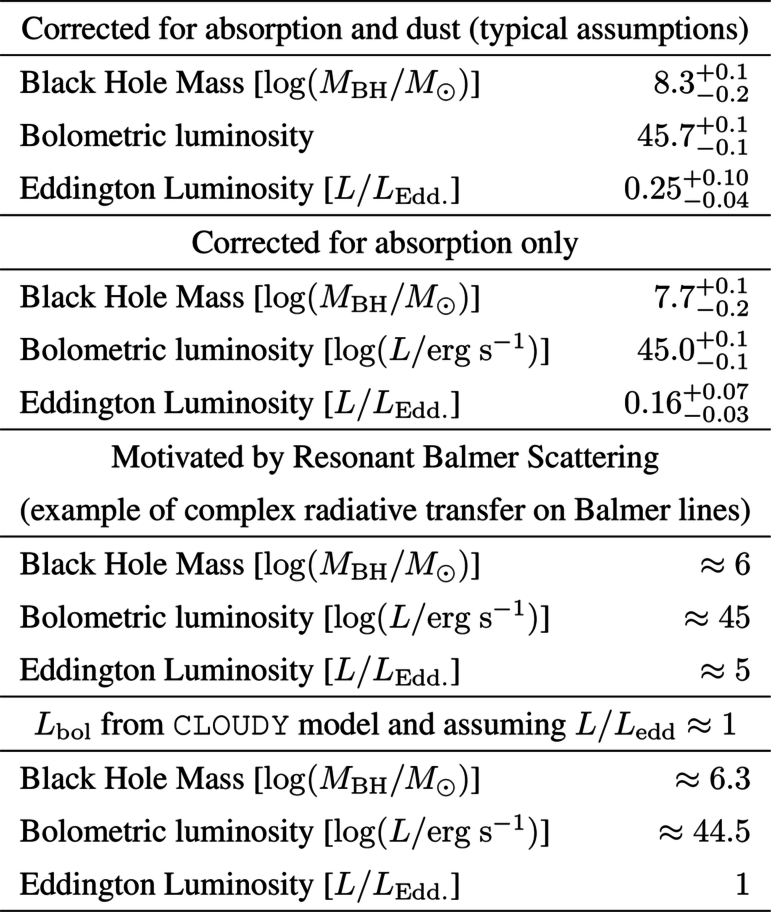
SMBH properties derived using local scaling relations based on H*β*

The key caveat is that it is unclear if these relations are applicable given the extraordinary physical conditions observed in this object. See section in text on SMBH properties for details.

## Data Availability

The prism spectra obtained as part of JWST-GO-5224 (MoM) featured in this work are available on Zenodo^86^ (10.5281/zenodo.15059214). All processed images and spectra used in this work are publicly available via the DAWN JWST archive (https://dawn-cph.github.io/dja/).

## Code availability

All results presented may be reproduced with the open-access reduced data described above and using the following publicly available software whose use is referenced in the text: msaexp, grizli, astropy, Cloudy, SpectRes, pysersic, COLT and numpyro.
