# Supplementary material for: A gas-enshrouded and gas-reddened black hole at cosmic dawn
Source: Nature. 2026 Aug 12;656(8127):329–33. doi: 10.1038/s41586-026-10846-4 (PMC13468124; doi:10.1038/s41586-026-10846-4)
Supplement: Supplementary file 1 [file 41586_2026_10846_MOESM1_ESM.pdf]

nature portfolio

Peer Review File

A Gas-Enshrouded, Gas-Reddened Black Hole at Cosmic Dawn

Corresponding Author: Dr Rohan Naidu

Any redactions in this file are there to maintain patient confidentiality, the confidentiality of unpublished data, or to remove third-party material.

Parts of this Peer Review File have been redacted as indicated to remove third-party material.

Attachments originally included by the reviewers as part of their assessment can be found at the end of this file.

Version 1:

Reviewer comments:

Referee #1

(Remarks to the Author)
The authors present here a somewhat interesting discovery, a very high-redshift AGN with a stronger Balmer break than usual. As the authors comment in their methodology section, the physical interpretation is still quite uncertain due to high degeneracies and limitations in the grid that has been employed for comparing the observed spectrum with detailed models.

As a main result, it is stated that the gas in the AGN is unusually dense, where number densities of the order of 10^11/cm^3 have been derived based on Cloudy models. However, even just with google it is easy to confirm that this is a rather typical number density for broad line regions of AGN.

Some implications are interesting, though overall I find the extrapolation from this single source to the overall population of Little Red Dots rather extreme. It is a possibility that similar processes occur in these sources, but evidence for that is not presented in the submitted manuscript. I believe rather than trying to derive some speculative conclusions for the overall population, it would be better to first aim to arrive at a solid understanding of this source and its properties. Particularly the absence of relevant signals of a stellar population is not discussed in any detail in the manuscript, and it could be a hint that perhaps what is detected here is a rather unique phase during the evolution of the galaxy, or at least that possibility should be discussed.

I also want to comment that I do not find the expression of a black hole star very appropriate. The authors comment themselves that what they model is essentially a supermassive black hole in an accretion disk, and they assume very large turbulent velocities, which are certainly not typical for a star. Perhaps the expression is meant to reflect the behaviour of the Balmer break that is known from stars; nonetheless the environment that is observed here is a highly dynamical one and calling it a star can give a highly misleading impression.

Finally, as the authors point out themselves, similar trends of Balmer absorption were reported already in their reference 15, including hints of very dense gas - which in the context of AGN is not particularly surprising in general. It is of course a relevant technological achievement that it can meanwhile also be detected at extremely high redshift.

I currently do not recommend this paper for publication in general, due to the reservations I outlined above. I believe that the general discussion should be improved including more quantitative comparisons both to the Little Red Dot population and even just to standard properties of broad line regions at low redshift, while avoiding too far-reaching and speculative conclusions about the entire population. I think that the source can be interesting, but perhaps the presentation of the results would benefit from a more neutral analysis without the pressure to achieve too far-reaching conclusions.

Referee #2

(Remarks to the Author)
This work reports an interesting study of the object MoM-BH*-1, at redshift z~7.75, which displays the strongest Balmer break of any known source across all redshifts. In addition, it has broad and symmetric double-peaked H-beta emission, and absorption in H-beta and H-gamma. Given the presence of broad emission lines and hints of variability, the authors model this source as an AGN. To explain the strength of the unusually large Balmer break, together with the absorption features and IR emission, they build upon the model of Inayoshi and Maiolino 2025 and argue that the central SMBH is embedded in a nearly dust-free environment with extremely dense and turbulent gas. They refer to this phase of the AGN as a "Black Hole Star", which may be a consequence of super-Eddington accretion. Further, they show that the symmetric double-peak of the H beta emission line implies that the surrounding gas acts as a static shell. They emphasize that the UV emission of the AGN is intrinsically weak due to the high opacity gas and not dust, and caution that applying steep dust corrections in such cases would lead to overestimates of the SMBH masses. They derive an upper limit on the host galaxy's mass assuming it to be the dominant contributor in the UV. Interestingly, they also report that the BH* is accompanied by a bright galaxy at around 60 proper kpc. They demonstrate that the combined spectrum of the system of bright galaxy and BH* resembles that of a little red dot (LRD), and propose that the perplexing features of LRDs across multiwavelength studies can be generally explained by a template of BH* embedded in brighter host galaxies.

Overall, the paper is very well-written, with a good discussion of the methods. The authors take care to exercise caution in their interpretation while not dismissing the possibility of alternate explanations. The nature of the source is unprecedented, which in my view makes it worth publishing in this journal.

I recommend the letter for publication following minor revisions and clarifications as outlined below:

Main text:

1. Figure 2: It would be helpful to explain why the uncertainty on the Balmer break strength for MoM-BH*-1 is significantly larger than for the other sources, and why it is asymmetric.
2. L161: I am slightly confused about the sentence on L/Ledd. Naively applying scaling relations should lead to a higher MBH and an underestimated L/Ledd, right? The values in parentheses seem to suggest the opposite.
3. Minor typo on L170: I think UV flux/radiation reads better
4. Figure 4a caption: The NIRCAM panels are not discussed in the caption
5. Figure 4b: Was the (LRD-host LRD) flux rescaled? Although difficult to tell just by eye, it appears that it has nearly the same BB strength as MoM-BH*-1.

Methods:
1. L 317: Perhaps cite Lin et al. (2024) for the 10% fraction of LRDs with Balmer breaks
2. Is there a reason to choose the prior range of H beta EW between 30 to 45 A, outside the measured 57 A?
3. Could the authors justify the choice of a uniform dust screen in post-processing? How would other geometries affect the results?
4. L434 & 454 typo: should be extended table 5
5. L457 typo: should be extended figure 5
6. If the BH* host is interpreted as quenched, but there is no detection of outflows, could AGN-driven feedback still be a plausible explanation for the quenching or are there other mechanisms being considered?
7. Would a more realistic intrinsic AGN SED model include photon trapping? This seems consistent with both the UV weakness and the super-Eddington scenario.
8. The authors note that turbulence is expected based on conclusions from Hopkins P.F. et al., but if I understand correctly their simulations refer to turbulence in disks. The gas modeled here appears to lie farther, outside BLR. Is there evidence for turbulence at these scales?
9. Also, how does turbulence affect the double peaked profile due to resonant scattering?
10. The authors note hints of variability for MoM-BH*-1. Since many LRDs seem to lack variability, how does this model accommodate such sources?

Referee #3

(Remarks to the Author)
This is a remarkable study that reports the discovery of a high redshift source showing the strongest Balmer break ever observed, about a factor of two higher than expected from evolved stellar populations. This feature can be explained by dense gas surrounding the AGN as advocated by some theoretical works. Here are my main concerns or suggestions.

- Authors make no effort in presenting multi-wavelength data which may strengthen their claim and is required to establish the exact nature of this source. Has this source been looked in radio, X-ray and even with ALMA to get host galaxy properties? I strongly encourage authors to present multi-wavelength observations.

- One of the broader implication of this study is that the proposed AGN scenario may explain larger population of LRDs and authors claim that Balmer scattering scenario may reduce the masses of SMBHs by ~ 2 orders of magnitude. This comes from the fact that broad lines do not trace BLR but scattering from medium outside. Consequently, intrinsic width of Balmer

lines would be narrower resulting in lower masses of SMBHs in LRDs. However, this scenario has been questioned by Joudzbalis et al. 2025 (arXiv:2504:03551) and can be tested in low redshift LRDs. They suggest that such scenario can be tested through Paschen lines, in particular with Pa-Beta which should trace intrinsic BLR emission due to its smaller scattering cross-section. They show in the case of GN-28074 that Pa-Beta has equally broader wings and invalidates the Balmer scattering scenario.

Authors should take LRDs data and show if Balmer scattering scenario is valid for larger population of LRDs. This should validate their scenario otherwise I'm afraid scope of this study is limited to this source. Also if masses of SMBHs are lower that would bring them below local MBH-sigma relation. How would authors reconcile this in the context of BH* and LRDs?

- The entire hypothesis of this study is the presence of dense gas in the vicinity of source producing Balmer break and also authors speculate that this may lead to super-Eddington accretion. This claim is supported by Ji et al. 2025 & Inayoshi & Maiolino et al 2025 (among others) but their Figs 2 & 3 show that at densities of 10^11 cm^-3, n=2 level saturates which results in weakening the strength of Balmer break for higher C> 0.5. How would authors reconcile this in the light of BH* observations.

-If BH is fed by dense gas, one would expect low ionization O-I emission lines likely produced by Ly-B florescence scenario (Inayoshi 2022b) would provide a good test and can probe super-Eddington accretion onto BH fed by dense gas. Why such lines are not observed for this source? Also I am wondering why semi-forbidden and permitted nebular lines such as NeIV, NIII,NIV, CIV and CII are not detected. Some of these lines lie in the realm of BLRs tracing extreme densities and have been observed in high z AGN such as GN-z11 in similar conditions (dense gas of > 10^9 cm^-3) (Bunker et al 2023, Maiolino et. al 2024 Nature).

- Authors arguments about super-Eddington accretion are bit speculative and require more evidence to claim this scenario. How come authors rule out the possibility of Eddington limited accretion?

Very minor points:

- Panels a, b & c are not marked in some Figs, "in the Table above", are you referring to ED table 4, please specificy it, line 547, EDF 5 not EFD2.

Version 2:

Reviewer comments:

Referee #1

(Remarks to the Author)
The authors have responded to my comments on the manuscript "A “Black Hole Star” Reveals the Remarkable Gas-Enshrouded Hearts of the Little Red Dots".

I want to first highlight that indeed there are some positive improvements in the new version of the manuscript. Particulaly Fig. 2b is useful to show the uniqueness of the sources that has been discovered. In principle the point could still be potentially made stronger and the figure could be improved by a relevant extend; for example for local galaxies and AGN I imagine that a lot more data must be available on the Balmer break strength including its statistical distibution and the frequency of some rare outliers, which potentially could help and contextualize the object that was found here. This will also help better to either demonstrate how unique the source really is, or allow us to understand it from what is known in the context of local AGN. In this sense an improvement was made but especially for the understanding of rare and unique sources, probably more could be learned by employing our knowledge from the already detected populations.

As part of their response, the authors argue that their statements concerning the wider LRD population are only presented as a "plausible theory" for the LRD population. While I believe this is true, that theory currently fills a quite relevant fraction of the manuscript and is implicitly implied by several of the figures in the draft, even though as also the authors say, there is no direct evidence for that from the analysis of additional sources. While of course a few lines on further implications could be ok, it is at least my impression that the presentation presents too much emphasys on something that is not established yet while remaining still somewhat superficial in analysing on how extreme and rare the source actually is.

What I also find to be missing is any attempt of explanation on why the properties of the object are so extreme. While the authors emphasize in their response on how unusual the strength of the Lyman break actually is, I believe the paper lacks an attempt to provide an astrophysical reason on how this could happen, and why this source is so different from many others. I believe that at least an educated guess on that would be needed before attempting any further-reaching conclusions.

Referee #2

(Remarks to the Author)
I thank the authors for their responses to my comments and for the revisions made to reflect them in the manuscript. I am happy with these. Indeed, one of the points I had been planning to follow up on regarding the similarity in Balmer break strength between MoM-BH* and host-subtracted LRDs has since been explored in a recent follow-up work that includes the present authors.
That said, the current manuscript already presents a well-supported result, and I congratulate the authors on this exciting work.

Referee #3

(Remarks to the Author)
-I requested that "Authors should take LRDs data and show if Balmer scattering scenario is valid for larger population of LRDs. This would validate their scenario otherwise I'm afraid scope of this study is limited to this source." Authors based on previous studies argue that "electron scattering imparts exponential wings to Paschen lines as well as Balmer lines that explains their similar appearance". However, the physical conditions required for electron scattering differ significantly from those required for Balmer scattering. The former requires highly ionized gas, whereas Balmer scattering operates in a predominantly neutral medium. How would authors reconcile both scenarios given the different required conditions. Moreover, the resonance scattering may produce strong deviations from Case B flux ratios but cannot broaden Hβ due to conversion to Paα. It is therefore difficult to envision a physical scenario in which both mechanisms operate simultaneously.

-In the case of electron scattering, the claim made in this work does not appear to be novel. A recent study by Rusakov et al. (2026), published in Nature, applied their cocoon model to an ensemble of LRDs and demonstrated that electron scattering can account for the observed line broadening. The authors should clearly distinguish their results from this existing work or clarify what new insight their study provides beyond the findings of Rusakov et al.

-A similar concern was raised by Referee #1, who noted: “I find the extrapolation from this single source to the overall population of Little Red Dots rather extreme.” Unfortunately, the authors have not made a substantive effort to address this issue. Rather than presenting observational evidence for a broader population of LRDs, they rely primarily on analytical arguments and interpretations drawn from previous studies. Indeed, the authors themselves acknowledge that “the main value of the paper is the discovery of the object,” rather than a demonstrated applicability of their conclusions to the LRD population as a whole. Given the lack of supporting evidence extending beyond this single source, the scope of the work remains limited. As such, while the discovery itself may be of interest, the manuscript does not provide sufficient justification for the broad claims required for publication in Nature. The study would be more appropriately placed in a specialized astronomy journal.

-The authors also appear to over-emphasize and over-interpret the BH* phenomenon by associating it with quasi-stars. This assumption significantly constrains their interpretation of the source properties and may bias their conclusions. A more comprehensive and balanced analysis would have been possible had the authors seriously considered alternative scenarios already discussed in the literature—such as those presented by Rusakov et al. (2025, Nature), Pacucci et al. (2026), and related works—and compared their findings accordingly. Such a comparison would have provided a more robust understanding of the nature of the source. It is unclear why the authors are reluctant to engage with these alternative explanations and instead strongly advocate for their own hypothetical BH* model. Exotic objects such as quasi-stars have never been observationally confirmed and are increasingly regarded as disfavored from the standpoint of modern stellar evolution theory. As a result, interpretations relying heavily on such objects are inherently uncertain. This concern has already been raised by one of the referees, who noted: “I also want to comment that I do not find the expression of a black hole star very appropriate.” Moreover, many of the quintessential stellar features highlighted by the authors can be naturally explained within the frameworks proposed in the aforementioned studies, without invoking quasi-stellar objects. Finally, it should be emphasized that the authors’ entire interpretation is based on one-dimensional CLOUDY models, which fall short of capturing the complexity of realistic astrophysical environments. In particular, the absence of turbulence—known to have significant implications for line broadening and black hole mass estimates—raises further concerns about the robustness of the derived conclusions.

-I reiterate my earlier concern, as the authors still do not present any concrete evidence to support their claims about super-Eddington accretion. which remains largely speculative and lack the quantitative justification required to substantiate this scenario. It is unclear on what basis the authors rule out the possibility of Eddington-limited accretion, which remains a viable and well-motivated alternative. Therefore, the conclusion in favor of super-Eddington accretion is not sufficiently supported.

-In conclusion, I am not convinced that the proposed BH* scenario can be extended to a larger—let alone the entire—population of LRDs. Several of the key arguments, as discussed above, remain speculative and would require additional observational evidence to robustly establish the nature of LRDs. As it stands, the data presented in the manuscript do not

justify such broad generalizations.

Version 3:

Reviewer comments:

Referee #1

(Remarks to the Author)
I thank the authors for the improvements compared to the previous version and for focusing the paper more strongly on the source that has been detected, which I believe merits investigation and discussion in its own right, and I also appreciate that more details on how this source compares to the typically known AGN have been added. Nonetheless I also have some remaining concerns about the presentation, that I want to line out here:

1) I previously expressed my discomfort with the name black hole star. The authors have argued that this name is mostly a figure of epic and prosaic speech, which in part can be true. However, it is still important that this concept is clearly defined and applied in a consistent way throughout the manuscript, also to still have clarity on what we mean by it within the community. Within the manuscript, the authors essentially say that they model a black hole within an accretion disk of gas, which I believe very likely is the correct description of what they are doing. On the other hand, in the introductory bold-face paragraph, they define it as a "theoretical configuration proposed to rapidly grow black holes via super-Eddington accretion", refering then to references 10-14, which are references mostly refering to so-called quasi-stars or supermassive stars, which are very different from the disk-like configurations that would be more plausible. I believe this is at least misleading and could very easily misinterpreted, and the authors should really quote papers that really refer to disk-type structures and remove the references that refer to quasistars that correspond to a very specific hydrostatic equilibrium configuration which really has not been investigated here. Such configurations have been proposed for very early stages when the supermassive black hole was about to form, not as a general configuration for a black hole in an accretion disk, and to my knowledge there are not even theoretical studies that would suggest such a configuration to form within an accretion disk. I also believe, in case the possibility of a quasistar is really considered, that the type of modeling should be different, including at least a 1D model of the stellar structure of the quasi-star together with an atmospheric model that determines on what would be visible. If the authors have ideas on this specific case, perhaps they should rather explain these in another part of the manuscript or provide further details in the supplementary material, while the core summary should focus on what is clearly established.

2) My previous question on the properties of the object that formed here has been possibly misunderstood. My interest was about how to generate an object with such an extreme Balmer break as found here and why it appears to happen so rarely. Is the explanation only the gas column density from the authors point of view? What does it then mean if in this particular object, such a high gas column density is present? Is it only a rather peculiar initial condition? Or do we see a very short-lived temporary phase, that may have existed also in other sources, but not long enough to be observed? I understand that perhaps it cannot yet be answered fully, but i think nonetheless the question still should be raised.

3) I believe there is also something to be learned from the argument by the authors that we see a somewhat lucky circumstance that the black hole star is forming in a faint source. This certainly can be so, but the question is why is it faint. Is it that the stellar population is rather old, or are there simply less stars in that source? Does it mean that the mass and luminosity of the new black hole star is usually similar, irrespective of the properties of the host galaxy? That would be a possible useful point to make, if it is what the authors indeed intend to express.

Referee #3

(Remarks to the Author)
-The authors state that “we agree that extrapolating from one source to the full population of LRDs is challenging and beyond the scope of this manuscript,” yet a substantial portion of the Methods section remains devoted to comparisons with LRDs, particularly on pages 10–11 under the headings “SMBH Properties and Ramifications for LRD Parameters” and “Host Galaxy Properties.” In fact, the terms “LRDs” and “Little Red Dots” appear nearly 40 times throughout the manuscript. This seems inconsistent with the statement in the referee response that “in this revision we have sharpened the focus on the unique nature of this source.” The manuscript should be revised more carefully to emphasize the discovery of this source and its specific properties. Any discussion of possible implications for LRDs should be brief and clearly framed as speculative, given that the authors do not provide evidence supporting conclusions about the broader LRD population. Additionally, Figure 4 appears to be largely speculative and does not represent a central result of this study. Therefore, It should be removed it from the manuscript.

-Related to the previous point, the abstract also contains speculative statements that should be removed. For example: “If the BH* merged with its brighter neighbor, it would resemble the recently discovered ‘Little Red Dots’” and “SMBH masses of such sources may therefore be overestimated by orders of magnitude.”

-The central premise of the BH-star scenario relies on the presence of a thick, dense gas envelope surrounding the black hole. The necessity of such an envelope appears to stem from attempts to reproduce the observed spectrum using Cloudy models. However, the authors do not explain how or under what physical conditions such an envelope would form, nor how long it could be sustained. The manuscript lacks a physical interpretation of this scenario, and relying on this assumption

without adequate justification is not sufficient.

- In their response, the authors state that the BH-star scenario is not based on quasi-stars, but more generally refers to a massive black hole enshrouded by dense gas, potentially including BHs in gas-rich stellar clusters, direct-collapse black holes forming in atomic cooling halos, or systems arising in the aftermath of galaxy mergers. However, after carefully reading the manuscript, I could not find a clear or consistent explanation of what is meant by the “BH-star” model in this broader context. The concept remains insufficiently defined. In addition, the literature review appears somewhat unbalanced. Most of the cited works relevant to this scenario focus on quasi-stars, which seems inconsistent with the authors’ claim that their model is not based on quasi-stars. A more representative and clearly justified selection of references is needed.

-The new title, “A Black Hole Star” does not adequately reflect the primary result of the paper—namely, the discovery of a unique source exhibiting the largest Balmer break. I recommend that the authors revise the title to better highlight this original finding.

As noted above, the “BH-star” scenario is neither uniquely defined nor newly introduced in this work, despite the impression given by the manuscript. Rather, it appears to be one of several possible interpretations, as the authors themselves state: “BH* as introduced in this work denotes the empirical phenomenon encompassing all channels.” In this context, the use of the term “BH*” is misleading. The authors should drop this notation and instead adopt a more general and established description, such as a black hole enshrouded by dense gas.

-The authors continue to place strong emphasis on the super-Eddington scenario, as reflected in the abstract and throughout the manuscript, while giving little attention to the possibility of Eddington-limited accretion. This imbalance should be addressed more carefully to provide a more complete and balanced discussion of the viable scenarios.

-A major caveat in the treatment of resonant scattering is the absence of turbulence in the shell models used within the COLT radiative transfer framework. On the one hand, the authors invoke a turbulent velocity of ~500 km/s (see Table 4) to reproduce the observed spectrum; on the other hand, turbulence is not included in the COLT model. This apparent inconsistency must be addressed, as it has significant implications for the conclusions of the present work and cannot be deferred to future studies.

Open Access This Peer Review File is licensed under a Creative Commons Attribution 4.0 International License, which permits use, sharing, adaptation, distribution and reproduction in any medium or format, as long as you give appropriate credit to the original author(s) and the source, provide a link to the Creative Commons license, and indicate if changes were made.
In cases where reviewers are anonymous, credit should be given to 'Anonymous Referee' and the source.
The images or other third party material in this Peer Review File are included in the article’s Creative Commons license,

unless indicated otherwise in a credit line to the material. If material is not included in the article's Creative Commons license and your intended use is not permitted by statutory regulation or exceeds the permitted use, you will need to obtain permission directly from the copyright holder.
To view a copy of this license, visit https://creativecommons.org/licenses/by/4.0/

Referee #1 (Remarks to the Author):

The authors present here a somewhat interesting discovery, a very high-redshift AGN with a stronger Balmer break than usual. As the authors comment in their methodology section, the physical interpretation is still quite uncertain due to high degeneracies and limitations in the grid that has been employed for comparing the observed spectrum with detailed models.

We thank Referee #1 for their healthy skepticism about the significance of this result. They have highlighted various areas where we can be clearer about the extraordinary nature of this source. We summarize Referee #1's key critiques as *"There is little of significance that distinguishes this object from AGN or other Little Red Dots. It is unclear what the broader implications are."* As our responses below will elucidate in detail, the comparison to standard broad-line regions and generic LRDs is quite clear – the BH* in MoM-BH*-1 stands alone, with unprecedented Balmer breaks and gas geometry that has no comparison. Even the extrapolation to the full LRD population may be natural – as per our proposal, many of their mystifying properties may be explained if their central engines are BH*s embedded in generic galaxies. Like Referee #2, we believe that *"the nature of the source is unprecedented, which in my view makes it worth publishing in this journal"* and in what follows hope to persuade Referee #1 that this is indeed, in Referee #3's words, "*a remarkable study"*.

To begin with, MoM-BH*-1 does not merely present a "*stronger Balmer break than usual"*. To the best of our knowledge, this is the strongest Balmer break observed in any object at any redshift (alongside the *Cliff*; de Graaff+25a). This is not just an academic distinction, but critical in the context of LRDs – Balmer breaks in these objects have been at the center of a major debate about whether these sources may be powered by massive galaxies/old stellar populations (e.g., Labbe+23). This paper marks a turning point. The break in this object is so strong – >2x stronger than the extant literature, and crucially, >2x stronger than the stellar population maximum or any individual star (Fig. 2; de Graaff+25a) – that it demands an extraordinary non-stellar explanation. In the typical LRD with a moderate break, this strong conclusion would be impossible and a myriad other solutions would be possible. Building on existing theoretical work, we have offered a potential explanation (the "Black Hole Star", "BH*" hypothesis).
Regardless of the modeling, that is clearly still in its infancy, in our opinion the main value of the paper is the discovery of the object alone and the new direction for models it has inspired (e.g., Liu+25, Kido+25, Begelman+25). As demonstrated in Figure 2, that this source is an empirical stand-out is clear. And then we have done our best to underscore that ours is far from the last word on the interpretation – in Referee #2's words, "*The authors take care to exercise caution in their interpretation while not dismissing the possibility of alternate explanations."*

As a main result, it is stated that the gas in the AGN is unusually dense, where number densities of the order of 10^11/cm^3 have been derived based on Cloudy models. However, even just with google it is easy to confirm that this is a rather typical number density for broad line regions of AGN.

We thank the referee for highlighting this area for improvement in clarity. The referee's point is of course true, dense gas is common among AGN. However, previously known AGN populations do not show such Balmer breaks or Balmer absorption. It is not only the density of the gas, but also the total column density and geometrical configuration (e.g., covering factor) that matter in producing this unprecedented Balmer break. In particular, the gas may be enveloping the black hole akin to a

stellar atmosphere such that the emergent spectrum now bears quintessential ``stellar" features (e.g., Balmer breaks, a black-body like continuum, stellar absorption-like features). This is a novel physical phenomenon and far from typical for broad-line regions. We now clarify this aspect in the paragraph where we introduce the term "Black Hole Star".

Some implications are interesting, though overall I find the extrapolation from this single source to the overall population of Little Red Dots rather extreme. It is a possibility that similar processes occur in these sources, but evidence for that is not presented in the submitted manuscript. I believe rather than trying to derive some speculative conclusions for the overall population, it would be better to first aim to arrive at a solid understanding of this source and its properties.

We thank the referee for raising this concern. We only seek to offer this as a plausible theory to explain the population, without concluding that this is *the* answer. Of course, this is a single source, but its mere existence is proof of a new class of objects (BH*s). With this proof of existence in hand, it is perhaps not a major leap to posit that the family of BH*s (with e.g., variations in luminosity/temperature/surface gravity) may occur not only in the very faint host galaxy like the one studied here, but also in other kinds of host galaxies (e.g., Fig. 4). Once this assumption is made, many of the key perplexing features of LRDs enumerated in the manuscript fall out naturally. For example, the perplexing V-shape SED (\ from the host, / from the BH*), the X-ray/IR-faintness (see the SED shape in Extended Data Fig. 3), the complex Balmer profiles (central emission from a host + wings/absorption from the BH*), and diversity in LRD SEDs (diversity in host to BH* ratio).

Particularly the absence of relevant signals of a stellar population is not discussed in any detail in the manuscript, and it could be a hint that perhaps what is detected here is a rather unique phase during the evolution of the galaxy, or at least that possibility should be discussed.

We thank the referee for flagging the importance of the stellar population. We stress that the rest optical continuum is ruled out with very high confidence to be arising from a stellar population due to the strength of the Balmer break that cannot be from stars. This leaves only the nebular emission and UV.

We are unsure how to highlight the stellar populations even further beyond the section dedicated to this issue at the moment ("Host Galaxy Properties"). We place constraints on the (very faint) stellar population that is being outshone by the BH* in this section. In particular, the [OIII] emission and perhaps some fraction of the UV emission are likely originating from the host. The [OIII] is narrow and has a critical density far lower than the gas density we infer for the BH* and hence likely originates from the host. The UV continuum may also partially arise from the host as per our best BH* model. However, there is little more we can infer about the evolutionary phase of the galaxy based on these two data points (upper limit on Muv, and 3-sigma [OIII] detection) – it may very well be a generic UV-faint galaxy with properties typical for this redshift. What this source does clearly demonstrate is that the BH* phenomenon can occur in extremely UV-faint (and perhaps low-mass/low-metallicity sources).

I also want to comment that I do not find the expression of a black hole star very appropriate. The authors comment themselves that what they model is essentially a supermassive black hole in an accretion disk, and they assume very large turbulent velocities, which are certainly not typical for a

star. Perhaps the expression is meant to reflect the behaviour of the Balmer break that is known from stars; nonetheless the environment that is observed here is a highly dynamical one and calling it a star can give a highly misleading impression.

We thank the referee for urging us to be clearer about the etymology. Of course, this source isn't literally a star (e.g., no nuclear fusion) and we hope it is clear to readers that we are exercising a reasonable degree of poetic license. We stress, however, that the scenario is remarkably analogous to the physics of stellar atmospheres, and there are deep insights that may be mined from this analogy. Along with the Balmer break, there are several other quintessentially stellar features we now highlight in the text that strengthens this point:

(1) Various authors have noted that the continuum SED of MoM-BH*-1 and sources of its ilk are remarkably blackbody-like, with a peak in the rest-optical and rapid fall off towards longer and shorter wavelengths (see e.g., Extended Data Figure 3; Lin+25, Ji+25, de Graaff+25b). This parallels the blackbody-like SEDs of ordinary stars but with much higher luminosities and far larger radii.

(2) The fact that the Balmer series *increases* in absorption strength as one goes down the series is remarkably reminiscent of stellar spectra (e.g., Hg in MoM-BH*-1 is in pure absorption in this source vs. Hb is still in emission). The generic case of an intervening cloud along the line of sight (instead of our proposed photosphere) would result in absorption EWs proportional to oscillator strengths (i.e., absorption in Hb>Hg). In a related vein, we note that other classic stellar absorption features (e.g., the Calcium triplet, Ca H&K, NaD, Fe forest) have now been reported in a growing number of LRDs (e.g., d'Eugenio+25, Lin+25, Torralba+25), which as per our proposed picture emanates in the atmospheres of BH*s.

The toolbox of stellar astrophysics (e.g., "fundamental stellar parameters", the HR diagram, the Hayashi track) is proving critical in understanding these objects (e.g., Santarelli+25, Kido+25, Begelman+25). For example, if these objects are truly radiating like stars, they could be fully described in terms of their effective temperatures (Teff), surface gravities (logg), composition ([Fe/H], abundances), and luminosity (L_bol) and must follow relationships such as the Stefan-Boltzmann law.

For all these reasons we find the term "BH*" to be apt. The community is now using this term regularly, with it already appearing in >45 publications as per ADS as a shorthand for our suggested scenario.

Finally, as the authors point out themselves, similar trends of Balmer absorption were reported already in their reference 15, including hints of very dense gas - which in the context of AGN is not particularly surprising in general. It is of course a relevant technological achievement that it can meanwhile also be detected at extremely high redshift.

We thank the referee for encouraging us to contextualize our discovery against known AGN. Virtually every LRD studied with R~3000 (NIRSpec/H grating) spectra shows Balmer absorption (though there could be a selection bias as the reddest sources with likely high gas columns tend to be chosen for follow-up). The absorber fraction (from lower-resolution spectra) is conservatively estimated to be >10-20% (see discussion in d'Eugenio+25; arXiv:2506.14870). On the other hand,

the fraction among pre-JWST AGN samples is <<0.1%, a remarkable ~100-1000x lower (e.g., Maiolino+25) with only a handful examples known (see the three papers cited in Matthee+24). It is interesting to note (also in reference to the etymology of "BH*") that Balmer absorption and line profiles with similarities to LRDs (e.g., exponential wings) are commonplace among a variety of stellar phenomena such as Type IIn supernovae (e.g., SN1994W; Dessart+09) and outbursts in luminous blue variables (e.g., η Carinae; Hillier+01) that involve a cocooned central engine.

But critically, the Balmer break accompanying Balmer absorption in MoM-BH*-1 is unprecedented. And while indeed we have ourselves discovered Balmer absorption in LRDs (Ref 15; Matthee, Naidu+24), it is only this remarkable object that has helped us tie the origin of the ubiquitous Balmer absorption to the physics of BH*s.

The extremely high redshift of the source is not merely a technical achievement but is deeply scientifically relevant. To explain how we end up with >1 billion solar mass black holes at z>6 already, we need to establish plausible progenitors and phases of extraordinary seeding or super-exponential growth at even earlier epochs. MoM-BH*-1 at z=7.76 (and the few other even higher-z Little Red Dots) may exemplify such an extraordinary phase. It is noteworthy that the number density of such objects remains high even out to z~9.3 (Taylor+25), marking them as excellent progenitor candidates.

I currently do not recommend this paper for publication in general, due to the reservations I outlined above. I believe that the general discussion should be improved including more quantitative comparisons both to the Little Red Dot population and even just to standard properties of broad line regions at low redshift, while avoiding too far-reaching and speculative conclusions about the entire population. I think that the source can be interesting, but perhaps the presentation of the results would benefit from a more neutral analysis without the pressure to achieve too far-reaching conclusions.

We once again thank the referee for taking the time to review this paper, and for their comments that have helped strengthen the clarity of this work. We assure the referee that we feel no "*pressure to achieve too far-reaching conclusions*". The only pressure we feel is in doing the utmost justice to the rare fortune of discovering a new astrophysical phenomenon. We are grateful for the referee's careful consideration of our work and have incorporated their suggestions to improve the neutrality and quantitative grounding of the analysis.

Referee #2 (Remarks to the Author):

This work reports an interesting study of the object MoM-BH-1, at redshift z~7.75, which displays the strongest Balmer break of any known source across all redshifts. In addition, it has broad and symmetric double-peaked H-beta emission, and absorption in H-beta and H-gamma. Given the presence of broad emission lines and hints of variability, the authors model this source as an AGN. To explain the strength of the unusually large Balmer break, together with the absorption features and IR emission, they build upon the model of Inayoshi and Maiolino 2025 and argue that the central SMBH is embedded in a nearly dust-free environment with extremely dense and turbulent gas. They refer to this phase of the AGN as a "Black Hole Star", which may be a consequence of super-Eddington accretion. Further, they show that the symmetric double-peak of the H beta*

emission line implies that the surrounding gas acts as a static shell. They emphasize that the UV emission of the AGN is intrinsically weak due to the high opacity gas and not dust, and caution that applying steep dust corrections in such cases would lead to overestimates of the SMBH masses. They derive an upper limit on the host galaxy's mass assuming it to be the dominant contributor in the UV. Interestingly, they also report that the BH is accompanied by a bright galaxy at around 60 proper kpc. They demonstrate that the combined spectrum of the system of bright galaxy and BH* resembles that of a little red dot (LRD), and propose that the perplexing features of LRDs across multiwavelength studies can be generally explained by a template of BH* embedded in brighter host galaxies.*

Overall, the paper is very well-written, with a good discussion of the methods. The authors take care to exercise caution in their interpretation while not dismissing the possibility of alternate explanations. The nature of the source is unprecedented, which in my view makes it worth publishing in this journal.

I recommend the letter for publication following minor revisions and clarifications as outlined below:

We are grateful to the referee for their kind words on the manuscript and their affirmation of the significance of this result. We thank them for their eagle-eyed attention to detail and for suggesting a series of thoughtful improvements across the manuscript. In what follows, we have pursued all their suggestions that we summarize below.

Main text:
1. Figure 2: It would be helpful to explain why the uncertainty on the Balmer break strength for MoM-BH-1 is significantly larger than for the other sources, and why it is asymmetric.*

The larger uncertainty in MoM-BH*-1 relative to other sources is a reflection of the relatively lower SNR in the bluer window (3620-3720 Angstrom; median SNR/pix of ~3). In other sources at similar redshift with weaker breaks the flux does not drop as drastically and the SNR is correspondingly higher (e.g., median SNR/pix~7 for an LRD at similar redshift, z~7). The break strength is skewed, again, because of the noisier blue window (median SNR/pix~3 vs. ~14 in the red). We calculate the break strength as a ratio of the median fluxes in these windows by generating samples from perturbing the errors. The denominator of the ratio has larger relative uncertainty, and so small downward fluctuations in the denominator create disproportionately large upward changes in the ratio. We have updated the caption in Figure 2 to reflect this.

2. L161: I am slightly confused about the sentence on L/Ledd. Naively applying scaling relations should lead to a higher MBH and an underestimated L/Ledd, right? The values in parentheses seem to suggest the opposite.

This is indeed confusing – we thank the referee for flagging this. We quote percentages in the first set of parentheses (18%) and then switch to a ratio in the next (factor of 5-10). We have now switched both these values to ratios.

3. Minor typo on L170: I think UV flux/radiation reads better

Fixed following the referee's suggestion.

4. Figure 4a caption: The NIRCAM panels are not discussed in the caption

We thank the referee for spotting this – we have rewritten the caption to refer to these panels.

5. Figure 4b: Was the (LRD-host LRD) flux rescaled? Although difficult to tell just by eye, it appears that it has nearly the same BB strength as MoM-BH-1.*

Indeed, both the compared host-subtracted spectra are renormalized in the same wavelength window for comparison – we now clarify this in the caption. Spurred by the referee's comment, we have measured the BB strength of the host-subtracted residual (i.e., the putative BH*) of the z=7.0 LRD and find a value that is similar to MoM-BH*-1 (~8). We now make note of this in the caption. This further supports our proposal for the existence of BH*s among typical LRDs, wherein the strong Balmer breaks of the BH*s are being diluted by light from host galaxies. We thank the referee for the suggestion with compliments for their sharp eye and sizing up the BB strength in a glance.

Methods:
1. L 317: Perhaps cite Lin et al. (2024) for the 10% fraction of LRDs with Balmer breaks

Thanks for the relevant reference – we have now included it in this line.

2. Is there a reason to choose the prior range of H beta EW between 30 to 45 A, outside the measured 57 A?

Thanks for flagging this – we have corrected this to read 'H-beta in *net* emission with 30 A<EW<45 A". While the emission component we fit is indeed 57 A, the total absorption is ~20 A and so the net emission falls in the 30 to 45 A range.

3. Could the authors justify the choice of a uniform dust screen in post-processing? How would other geometries affect the results?

State-of-the-art modeling of dust attenuation in high-redshift galaxies (e.g., Shivaei+25) often invokes a two-component model – one component for the birth cloud enshrouding young stars, and another for a uniform screen representing the overall ISM that reddens both young and old stars. For the BH*, we ignore the equivalent of the birth cloud (motivated by the remarkable FIR weakness of LRDs) and apply only the uniform screen representing the galaxy ISM. Our best fitting models have negligible Av, and so our results have minimal dependence on the exact details of the dust geometry. We note that the low Av is also expected given the low-mass (and hence low-metallicity) of the almost invisible host. We have now added a note in the text to address the geometry.

4. L434 & 454 typo: should be extended table 5
5. L457 typo: should be extended figure 5

Fixed, thank you!

6. If the BH host is interpreted as quenched, but there is no detection of outflows, could AGN-driven feedback still be a plausible explanation for the quenching or are there other mechanisms being considered?*

We thank the referee for highlighting this conundrum. Indeed, in a BH* scenario, strong outflows/jets (which would disrupt the envelope) are not expected (e.g., Kido+25). Furthermore, essentially no known LRD shows outflow signatures such as broad [OIII]. And so the mechanism (if any) that may contribute to quenching remains unclear. We also note that at these low masses, the hosts are perhaps ``mini-quenched" and may currently be ``napping" in between bursts of star-formation. In this regime of inherently stochastic star-formation, the BH* may have nothing to do with the presently depressed star-formation rate of its host. We have made note of these nuances in the *Host Galaxy Properties* section.

7. Would a more realistic intrinsic AGN SED model include photon trapping? This seems consistent with both the UV weakness and the super-Eddington scenario.

The conditions of the BH* envelope (e.g., optical thickness) would indeed make it a natural environment for photon trapping. We have now mentioned this in the text as a possible effect that our simplistic SEDs do not explicitly capture at the moment. However, we note that given the large, diverse grid we explore, UV-weak models (e.g., with very low alpha_UV) have been tested as well and are the ones that work the best for reproducing the spectrum.

8. The authors note that turbulence is expected based on conclusions from Hopkins P.F. et al., but if I understand correctly their simulations refer to turbulence in disks. The gas modeled here appears to lie farther, outside BLR. Is there evidence for turbulence at these scales?

We thank the referee for highlighting this nuance – turbulence at larger scales, and in these envelope-like enormous structures is yet to be demonstrated in a realistic simulation. We clarify this distinction in the text and draw on Hopkins+ only to justify that at least in the inner regions there is some theoretical precedence for these high Mach numbers.

9. Also, how does turbulence affect the double peaked profile due to resonant scattering?

Broadly speaking, turbulence would make the velocity field (and line profiles) far more complex than assumed in our simple proof of concept shell model presented at the moment – e.g., photons may struggle to escape at the line center, and the wings could be further broadened (by analogy with Lyman-alpha – e.g., Section 4.5 and Figure 7 of Garel+24). We have updated the text to mention this as an important effect to account for in order to be self-consistent with the CLOUDY results, but defer the significant model development required to carefully characterize this to future work.

10. The authors note hints of variability for MoM-BH-1. Since many LRDs seem to lack variability, how does this model accommodate such sources?*

We thank the referee for encouraging us to contextualize this point against the literature. A possibility we have highlighted in this section is that a ``pure" BH* that is undiluted by light from its host galaxy may display a clearer variability signal than a generic LRD where the rest-optical light is a mixture

from host+BH*. Intriguingly, one of the handful of LRDs with a hint of variability (in its Balmer line EW) reported does have among the strongest Balmer breaks in the LRD population (Furtak+25). The break strength may be treated as a proxy for BH* dominance in the rest-optical as per the composite picture we have put forward here. We have made note of this point in the "Hints of Variability" section.

Referee #3 (Remarks to the Author):

This is a remarkable study that reports the discovery of a high redshift source showing the strongest Balmer break ever observed, about a factor of two higher than expected from evolved stellar populations. This feature can be explained by dense gas surrounding the AGN as advocated by some theoretical works. Here are my main concerns or suggestions.

We thank the referee for their kind description of this paper and recognition of the remarkable nature of this source. We are grateful for their thoughtful suggestions and for their encouragement to strengthen the connections to the full LRD sample. We have pursued all of their insightful suggestions as detailed below.

- Authors make no effort in presenting multi-wavelength data which may strengthen their claim and is required to establish the exact nature of this source. Has this source been looked in radio, X-ray and even with ALMA to get host galaxy properties? I strongly encourage authors to present multi-wavelength observations.

Upon the referee's suggestion we have searched existing multi-wavelength datasets for coverage of this source. The X-UDS Survey (Kocevski+18) has surveyed this field with *Chandra*. MoM-BH*-1 is not detected in these data and is indeed absent from the survey's source list. The non-detection translates to an upper limit on the X-ray luminosity of log(Lx/[erg/s]) = 44.5 at 5-90 keV (rest-frame) based on the survey's nominal depth that we now quote in the paper. We did not find any archival ALMA or radio data at the source's location.

We note that LRDs as a general population are extremely X-ray weak as well as radio/FIR weak (e.g., Greene+25, Casey+25). We would expect MoM-BH*-1 to follow this trend as well. Indeed, a key feature of the model presented in this paper is that effectively all the bolometric luminosity emerges in the rest-optical (Extended Data Fig. 3; see also Greene+25). We have expanded the caption of this figure and the discussion of the bolometric luminosity to highlight this.

As for the host galaxy, it would be quite challenging for ALMA to detect such a low-mass galaxy in a blank field unassisted by gravitational lensing. To make this clear, we now also include our best empirical estimate of the stellar mass of log(M*/M_sun) = 7.4^{0.6}_{-0.3} that follows from the allowed M_UV range. Previously we only quoted a 95% limit of log(M*/M_sun) <8.5 at z=7.76 as a maximally conservative upper limit.

- One of the broader implication of this study is that the proposed AGN scenario may explain larger population of LRDs and authors claim that Balmer scattering scenario may reduce the masses of SMBHs by ~ 2 orders of magnitude. This comes from the fact that broad lines do not trace BLR but

scattering from medium outside. Consequently, intrinsic width of Balmer lines would be narrower resulting in lower masses of SMBHs in LRDs. However, this scenario has been questioned by Joudzbalis et al. 2025 (arXiv:2504:03551) and can be tested in low redshift LRDs. They suggest that such scenario can be tested through Paschen lines, in particular with Pa-Beta which should trace intrinsic BLR emission due to its smaller scattering cross-section. They show in the case of GN-28074 that Pa-Beta has equally broader wings and invalidates the Balmer scattering scenario. Authors should take LRDs data and show if Balmer scattering scenario is valid for larger population of LRDs. This should validate their scenario otherwise I'm afraid scope of this study is limited to this source. Also if masses of SMBHs are lower that would bring them below local MBH-sigma relation. How would authors reconcile this in the context of BH and LRDs?*

We thank the referee for raising this critical set of interconnected issues that we address below. In summary, we are now far more confident about our interpretation that the black hole masses based on local AGN calibrations are overestimated. We are also increasingly convinced that the scattering mechanisms brought to light by MoM-BH*-1 might apply to the full LRD population.

The paper referenced by the referee (Juodzbalis+25; arXiv:2504:03551) has cast aspersions on scattering scenarios in LRDs based on the seemingly intuitive argument that a Paschen line in one low-z source has a similar line-shape as the Balmer lines. Fortunately, we can now refer to the detailed radiative transfer simulations for Balmer and Paschen lines in BH*-like scattering atmospheres presented by Chang+25 (arXiv:2508.08768). In particular, Chang+25 highlight two key mechanisms likely at play in the LRDs: (1) electron scattering that imparts exponential wings of comparable FWHM across Balmer and Paschen lines (see also Rusakov+25); (2) resonant Balmer scattering (that is responsible for the double-peaked profile in MoM-BH*-1). Both these processes in concert explain the line profiles of sources like MoM-BH*-1 and the LRDs. The critical implication is that line profiles no longer trace virial kinematics, but are set by the properties of the scattering medium

The objection raised by Juodzbalis+25 is fully addressed by this picture – electron scattering imparts exponential wings to Paschen lines as well as Balmer lines that explains their similar appearance. Brazzini+25 (arXiv: 2507.08929) have questioned the applicability of electron scattering by noting that the line widths aren't *exactly* the same – this can be explained by the lines forming at different depths encountering a diversity of temperatures and electron column densities (see e.g., Torralba+25 and Kokorev+25 for tomography of BH*s illustrating this point).

Now to the next issue – how can we prove that resonant Balmer scattering is occurring in the entire LRD population and not just in MoM-BH*-1? A critical implication of resonant Balmer scattering highlighted by Chang+25 is that the Balmer decrement in this scenario far exceeds the Case-B value (Ha/Hb>>2.86, reaching ~10 for our inferred gas densities in MoM-BH*-1). This is partly because H-beta photons scatter into Paschen-alpha and H-alpha, greatly enhancing the PaA/Hb and Ha/Hb ratios over the Case-B baseline. It is now well-established that LRDs show extreme Balmer decrements, with a typical value of ~10 (e.g., the population studies in Brooks+25, de Graaff+25b). A key implication of our BH* model (and stringent FIR non-detections; Casey+25) is that LRD SEDs are red not due to dust, but due to gas. A plausible explanation for the extreme Balmer decrements consistent with all other data pointing to such gas conditions/geometry at the moment is therefore resonant Balmer scattering. Hence, the scope of our study may not just be limited to a single source,

but may extend to the entire LRD population whose Ha/Hb~10 may be a hallmark of resonant Balmer scattering.

Further supporting our point about overestimated black hole masses, we now have a new fully self-consistent approach to estimating the bolometric luminosity. In particular, by simply integrating the best-fit CLOUDY model shown in Extended Data Figure 3, we find a value of L_bol = 10^44.5 erg/s that we report in Extended Data Table 5. This simple calculation already highlights the inapplicability of local scaling relationships – using them verbatim as done in the extant literature would produce a ~10x higher L_bol (see also Greene+25 for a detailed exploration of this issue). If the L_bol scaling from local AGN does not hold, this casts aspersions on the M_BH calibrations as well. Furthermore, we note that virtually all theoretical models in the literature that are able to broadly reproduce the key features of LRDs (such as deep Balmer breaks) invoke an envelope feeding a black hole at a super-Eddington or close to Eddington rate (e.g., Kido+25, Liu+25, Begelman+25). Inspired by these models, we may then estimate a black hole mass for the source assuming L_bol = L_edd which yields ~10^6.3 M_sun, further making the point that the standard approach would produce a ~100x overestimated mass (~10^8.3 M_sun).

Finally, the referee raises the point that if BH masses are indeed being overestimated by ~100x, correcting for this effect might bring previously “overmassive” sources *below* the local M_star-M_BH relation. First, we point out that host/AGN decomposition for LRDs remains extremely challenging given that we are only now beginning to understand that these AGN may be a new class of objects, i.e., BH*s. Therefore, *both* M_star and M_BH reported in the literature remain systematically uncertain, and the location of sources in this diagram may not be a robust diagnostic. Next, let us consider MoM-BH*-1: our upper limit for the stellar mass (assuming all the UV arises from a host) is <10^8.5 M_sun. For a BH mass of ~10^6.3 M_sun this yields an M_BH/M_star of <0.6%, consistent with the local ratio of ~0.01% (e.g., Reines & Volonteri 2015). Finally, the average M_BH/M_star reported in JWST studies of faint AGN based on local calibrations is of the order ~1-10% (see Fei+25 for a recent compilation and the median locus). Correcting this by two orders of magnitude brings this in agreement with, and not below the local relation.

We have incorporated these points in the text (in the *Resonant Balmer Scattering?* and *SMBH Properties* sections), and thank the referee for their encouragement to flesh out these details.

- The entire hypothesis of this study is the presence of dense gas in the vicinity of source producing Balmer break and also authors speculate that this may lead to super-Eddington accretion. This claim is supported by Ji et al. 2025 & Inayoshi & Maiolino et al 2025 (among others) but their Figs 2 & 3 show that at densities of 10^11 cm^-3, n=2 level saturates which results in weakening the strength of Balmer break for higher C> 0.5. How would authors reconcile this in the light of BH observations.*

We thank the referee for raising this point that may cause confusion. Figs. 2 & 3 of Inayoshi & Maiolino et al. 2025 plot the Balmer break strength as a function of gas density keeping all other parameters (e.g., column density, ionization parameter, SED shape) fixed – for this set of parameters there is a “sweet spot” in the gas density where the Balmer break strength is maximized. However, where this sweet spot occurs is a complex function of all the remaining parameters as well (which are different in our model relative to theirs). Indeed, in our grid we find that strong Balmer breaks may be achieved for diverse combinations of parameters (including the 10^11 cm^-3 in our best

model) that are able to sustain sufficient n=2 populations. We now show this explicitly by adding a new Figure (appended below) plotting the n=2 level density and emergent spectra as a function of depth in the cloud that should give readers a vivid feel for the model.

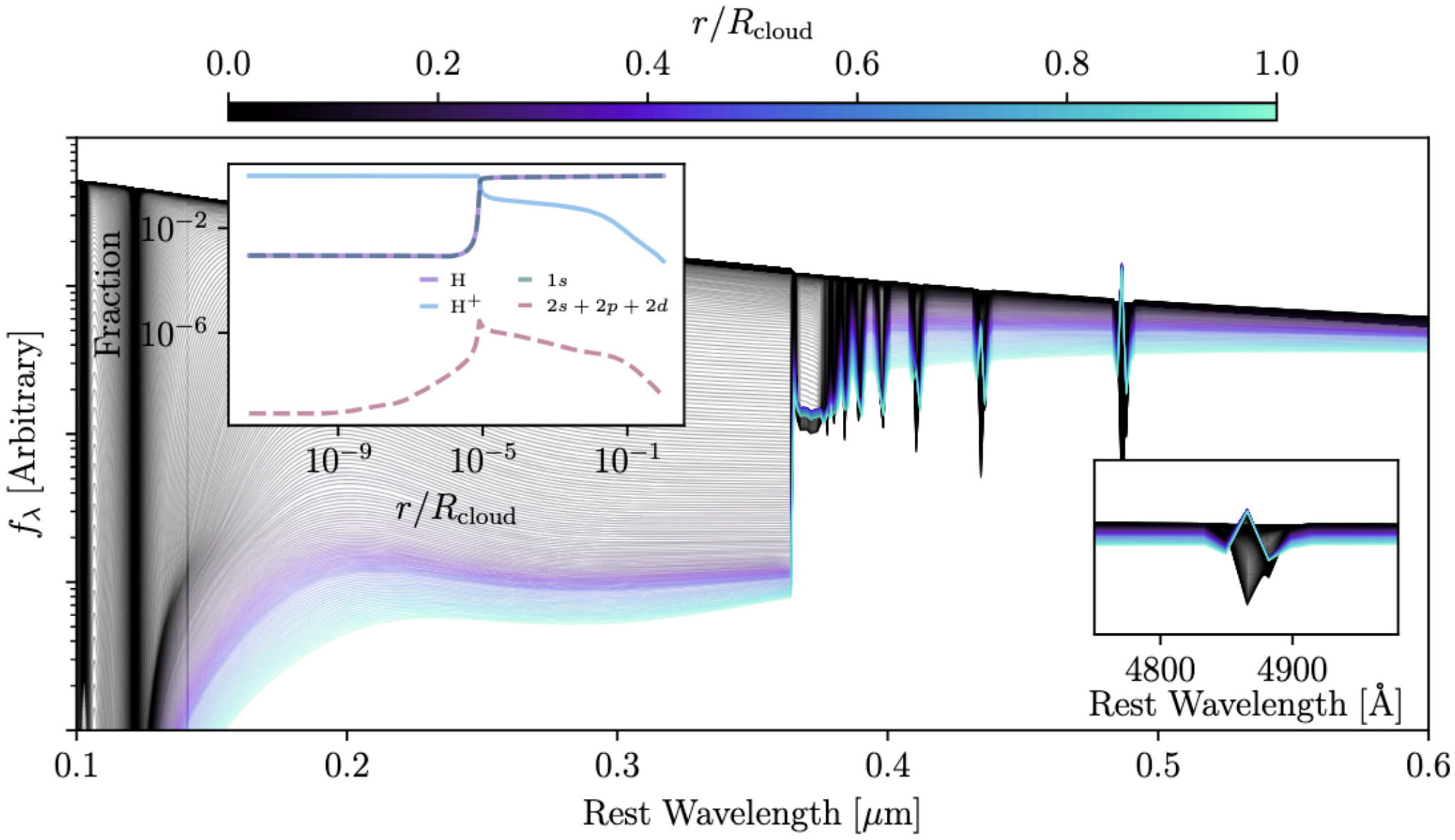

Extended Data Figure 4 | Cross-sectional view of the best-fit CLOUDY model. Emergent spectra as observed at various depths are overlaid in the main panel – the incident power-law continuum (black, $r/R_{\rm cloud} \approx 0$) is reprocessed such that a deep Balmer break and absorption features are imparted (light blue, $r/R_{\rm cloud} \approx 1$). The inset in the center shows the fractional density of ionized gas (H+), neutral gas (H), and the $n = 1$ and $n = 2$ levels – crucially, a significant $n = 2$ population exists to produce deep Balmer absorption. The bottom-right panel shows where the observed Hβ line arises from – remarkably, Hβ in emission is produced close to the surface (e.g., due to collisional effects) and has little to do with the kinematics of the interior – this has critical implications for BH mass estimates based on Balmer lines.

We also note that recent papers make this point about the diverse conditions that can produce a strong Balmer break – Liu+25 (arXiv:2507.07190; their Fig. 2) demonstrate variations in the Balmer break strength as a function of not only gas density but also temperature (i.e., the ionizing source properties in our context). Torralba+25 (arXiv:2510.00103; their Fig. 11) similarly show a high n=2 occupancy fraction for gas densities very similar to our best model (~10^10.5 cm^-3).

Another relevant point to the black hole mass estimates (discussed earlier above) is highlighted by this new figure. The Hb emission as per this model arises almost entirely from the *surface* of the cloud (e.g., via collisional excitation), not the inner regions. Which is to say, the Hb has little to do with the kinematics of a broad-line region (i.e., if it exists). Of course, Hb likely also still escapes from the inner regions via electron scattering wings and resonant scattering despite the high optical depth at line-center (as in the models of Chang+25 and our toy model discussed above), but nonetheless, this is yet another reason local calibrations for black hole masses should be treated with caution.

-If BH is fed by dense gas, one would expect low ionization O-I emission lines likely produced by Ly-B florescence scenario (Inayoshi 2022b) would provide a good test and can probe super-Eddington accretion onto BH fed by dense gas. Why such lines are not observed for this source? Also I am wondering why semi-forbidden and permitted nebular lines such as NeIV, NIII,NIV, CIV and CII are not detected. Some of these lines lie in the realm of BLRs tracing extreme

densities and have been observed in high z AGN such as GN-z11 in similar conditions (dense gas of > 10^9 cm^-3) (Bunker et al 2023, Maiolino et. al 2024 Nature).
- Authors arguments about super-Eddington accretion are bit speculative and require more evidence to claim this scenario. How come authors rule out the possibility of Eddington limited accretion?

We thank the referee for suggesting we look into these diagnostic lines to strengthen the case for super-Eddington accretion. Intriguingly, OI is indeed one of the most prominent features in low-redshift LRD spectra when transitions of this line are still within NIRSpec's reach (de Graaff+25b). For instance, Kokorev+25 recently analyzed OI in detail using the deepest medium-resolution LRD spectrum taken till date (~80h adjusted for lensing) in a z=3.5 source. Unfortunately, the most prominent OI lines seen in this object (8448 A, 11290 A) and other similar low-z LRDs (e.g., Wang+24) fall at wavelengths beyond NIRSpec's reach at z=7.76. The OI line at 1304 A occurs in a very low SNR region of the spectrum that is extremely faint below the Balmer break.

Nonetheless, following our argument that LRDs may be a combination of a BH* embedded in a host galaxy, the ubiquity of strong OI lines in LRDs, as the referee points out (Inayoshi+22b), provides circumstantial evidence for super-Eddington accretion in MoM-BH*-1. Further support for super-Eddington accretion comes from theory – all extant literature models able to explain the deep Balmer breaks and other peculiar features of BH*s invoke close to Eddington or super-Eddington accretion (e.g., Kido+25, Begelman+25, Liu+25). We now incorporate these points in the "SMBH properties" section of the text.

The UV lines suggested by the referee would be remarkably sensitive diagnostics as in the case of GNz11. Unfortunately, due to the extreme UV faintness of the source and the resolution of the prism we are unable to say anything definitive about these lines and line ratios from the current spectrum. We have forced emission line fits at their expected wavelengths but are unable to recover anything at >2 sigma. Deeper M/H-grating spectroscopy in future cycles of JWST observations are required.

Very minor points:
- Panels a, b & c are not marked in some Figs, "in the Table above", are you referring to ED table 4, please specificy it, line 547, EDF 5 not EFD2.

Fixed, thank you!

Referee #1 (Remarks to the Author):

The authors have responded to my comments on the manuscript "A “Black Hole Star” Reveals the Remarkable Gas-Enshrouded Hearts of the Little Red Dots".

I want to first highlight that indeed there are some positive improvements in the new version of the manuscript. Particulaly Fig. 2b is useful to show the uniqueness of the sources that has been discovered. In principle the point could still be potentially made stronger and the figure could be improved by a relevant extend; for example for local galaxies and AGN I imagine that a lot more data must be available on the Balmer break strength including its statistical distibution and the frequency of some rare outliers, which potentially could help and contextualize the object that was found here. This will also help better to either demonstrate how unique the source really is, or allow us to understand it from what is known in the context of local AGN. In this sense an improvement was made but especially for the understanding of rare and unique sources, probably more could be learned by employing our knowledge from the already detected populations.

We thank the referee for their kind words. We prefer to retain Figure 2 as is for clarity, and to focus on the high-redshift Universe that is relevant to the question of the emergence of early supermassive black holes. However, following the referee’s suggestion we have added two salient comparisons to the text to contextualize the object against local samples.

First, we point out that <<0.1% of AGN show Balmer absorption in the local Universe that is seen prominently in MoM-BH*-1 as well as LRDs (as discussed in Matthee+24, Maiolino+25; e.g., Hall+07) .

Next, we note that no object in the SDSS-DR16 AGN sample has a Balmer break within a factor of ~3x of the source under consideration. In particular, we measured the Balmer break distribution of the high-quality (SNR>10 in the redder Balmer break window) SDSS-DR16 AGN sample (Lyke+20). The median Balmer break strength is <1 (0.9+/-0.1) as expected from the typical power-law shape of AGN spectra. The objects with the deepest Balmer breaks have a strength of ~2.5 – these are sources where the AGN occurs in a massive quiescent galaxy (e.g., SDSS J012630.15-001151.3 at z=0.2396 with a Balmer break strength of 2.5+/-0.1) consistent with the stellar population maximum shown in Fig. 2b. In contrast, MoM-BH* has a break strength of 7.7^{+2.3}_{-1.4}.

As part of their response, the authors argue that their statements concerning the wider LRD population are only presented as a "plausible theory" for the LRD population. While I believe this is true, that theory currently fills a quite relevant fraction of the manuscript and is implicitly implied by several of the figures in the draft, even though as also the authors say, there is no direct evidence for that from the analysis of additional sources. While of course a few lines on further implications could be ok, it is at least my impression that the presentation presents too much emphasys on something that is not established yet while remaining still somewhat superficial in analysing on how extreme and rare the source actually is.

We thank the referee for their remarks. We agree that extrapolating from one extreme source to the population of LRDs is challenging. The importance of this paper is in fact the extreme nature of the

object, which proved without a doubt that the Balmer break cannot be stellar, whereas in all other prior sources this could not be concluded.

As we illustrate in Figure 4, this leads to the intriguing possibility that the diversity in LRDs is related to different compositions of hosts + BH*s. Obviously, this requires population level studies and detailed correlations between properties that are beyond the scope of this paper.

However, we believe this is an important concept to explore, not envisioned before, and follows directly from the extreme nature of the source. Many groups have since been building on our proposal in detail (e.g., Taylor+25, Golubchik+25, de Graaff+25b, Umeda+25, Yanagisawa+26, Sun+26, Barro+26, Wang+26), thereby showcasing the value and impact of presenting it here, even as a possible theory. Following the referee's suggestion we have significantly shrunk the final section of the manuscript and halved Figure 4 to focus fully on the simple empirical exercise and on data pertaining purely to the source of study.

What I also find to be missing is any attempt of explanation on why the properties of the object are so extreme. While the authors emphasize in their response on how unusual the strength of the Lyman break actually is, I believe the paper lacks an attempt to provide an astrophysical reason on how this could happen, and why this source is so different from many others. I believe that at least an educated guess on that would be needed before attempting any further-reaching conclusions.

We thank the referee for raising this point. There are two aspects to this issue. How do BH*s form in the first place? And why is the host galaxy so weak that the BH* light is observed essentially undiluted in this source?

We now cite several possible theories for how exactly BH*s form including e.g., entrapment in gas within a dense nuclear star cluster (Alexander & Natajan 2014), super-Eddington flows around a black hole (Liu+25), failed supernovae of supermassive stars (Begelman & Coughlin 2025). We believe the unique value of this paper is in empirically describing a novel astrophysical phenomenon (BH*s) – establishing how exactly BH*s form is a subject of very active research and beyond the scope of this paper.

As for the weakness of the host galaxy, the exercise in Fig. 4 suggests this may be a selection effect in the sense that we are perhaps fortuitously catching a BH* form in a very faint source. BH*s almost certainly occur in much more luminous objects as well, but in those objects they would produce V-shaped SEDs as in classical LRDs (as shown in Fig. 4) or be effectively invisible to us (see Figs. 7, 8 of the spatially resolved LRD case study in Yanagisawa+26 enabled by gravitational lensing; see also Fig. 15 of Sun+26 who demonstrate BH*+Host composites with varying relative fractions). Testing this hypothesis would require probing statistical correlations on large samples of such "pure" BH*s (e.g., if it is purely chance outshining, there should be no correlations between host and BH* properties) that do not exist at the moment. We now highlight this as an avenue for future work while ending the host galaxy properties section.

Referee #2 (Remarks to the Author):

I thank the authors for their responses to my comments and for the revisions made to reflect them in the manuscript. I am happy with these. Indeed, one of the points I had been planning to follow up on regarding the similarity in Balmer break strength between MoM-BH and host-subtracted LRDs has since been explored in a recent follow-up work that includes the present authors. That said, the current manuscript already presents a well-supported result, and I congratulate the authors on this exciting work.*

We thank the referee for their kind words. For the benefit of the editors and other reviewers – the paper being referenced here is Sun+26 that demonstrates the basic framework first proposed in this paper (Little Red Dot = Host Galaxy = Black Hole Star) appears to hold for the full population of LRDs.

Referee #3 (Remarks to the Author):

-I requested that "Authors should take LRDs data and show if Balmer scattering scenario is valid for larger population of LRDs. This would validate their scenario otherwise I'm afraid scope of this study is limited to this source." Authors based on previous studies argue that "electron scattering imparts exponential wings to Paschen lines as well as Balmer lines that explains their similar appearance". However, the physical conditions required for electron scattering differ significantly from those required for Balmer scattering. The former requires highly ionized gas, whereas Balmer scattering operates in a predominantly neutral medium. How would authors reconcile both scenarios given the different required conditions. Moreover, the resonance scattering may produce strong deviations from Case B flux ratios but cannot broaden Hβ due to conversion to Paα. It is therefore difficult to envision a physical scenario in which both mechanisms operate simultaneously.

The referee's key point is "*the physical conditions required for electron scattering differ significantly from those required for Balmer scattering".* Indeed, we agree with the referee's assessment. However, please note that these physical conditions do occur simultaneously in our proposed model. The medium is partially ionized, and the high density thus enables high opacity for both electron and resonant scattering. As we demonstrate in the "tomography" of the CLOUDY model included in the updated manuscript, there are abundant electrons (H+) as well as partially ionized gas with *n=2* populations (*2s+2p+2d*) throughout the structure. Therefore, the deviation from the Case B fluxes and exponential profiles are simultaneously observed within this picture. The broadening of H-beta relative to Paschen-alpha should also be thought of as follows – if one were to fit a Gaussian+absorber to the double peak H-beta profile, one would get a some FWHM that is far larger than the intrinsic line-width before these scattering processes.

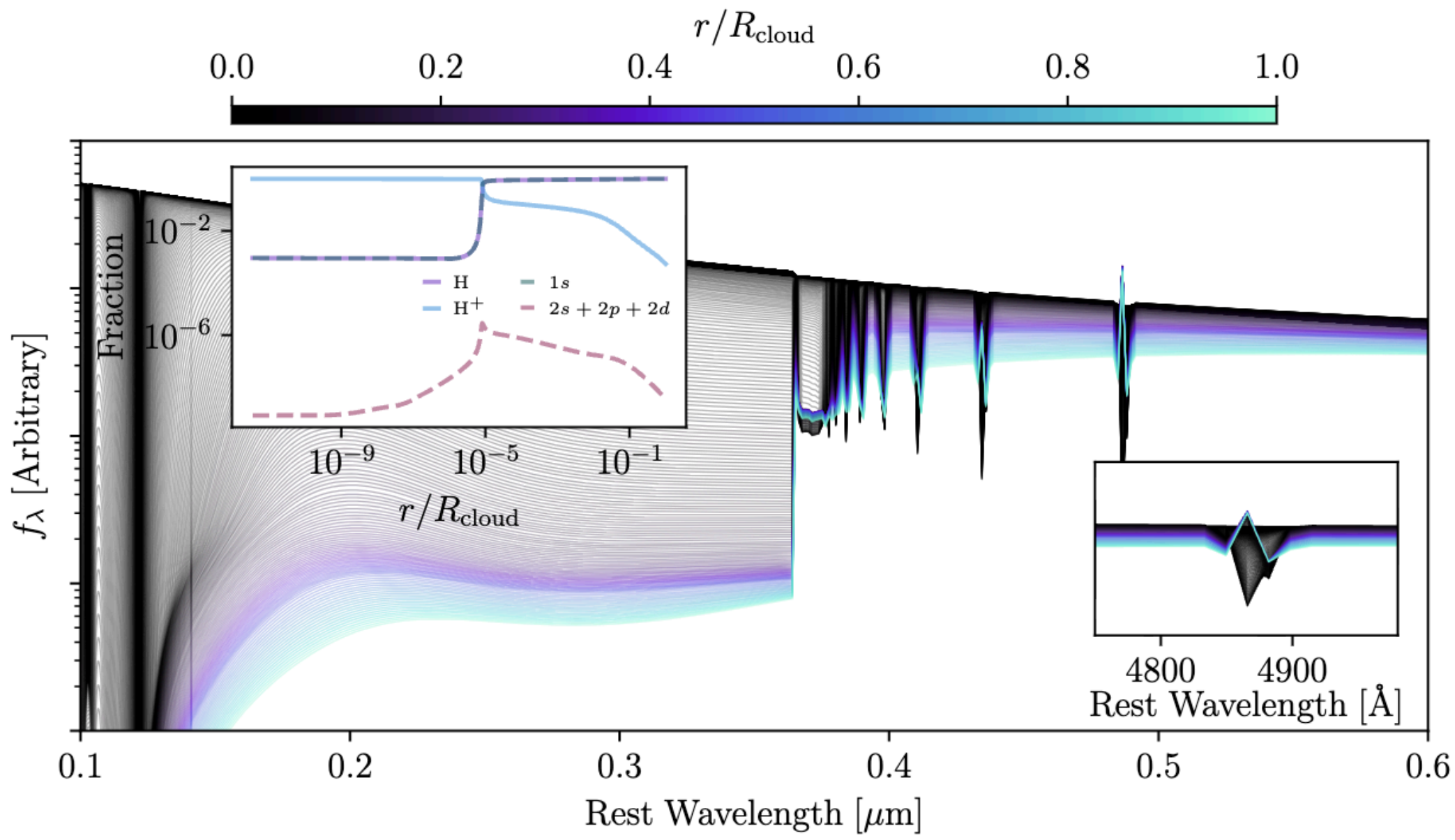

Extended Data Figure 4 | Cross-sectional view of the best-fit CLOUDY model. Emergent spectra as observed at various depths are overlaid in the main panel – the incident power-law continuum (black, $r/R_{\rm cloud} \approx 0$) is reprocessed such that a deep Balmer break and absorption features are imparted (light blue, $r/R_{\rm cloud} \approx 1$). The inset in the center shows the fractional density of ionized gas (H+), neutral gas (H), and the $n = 1$ and $n = 2$ levels – crucially, a significant $n = 2$ population exists to produce deep Balmer absorption. Note that there is no dust in the model, and we apply dust only as a post-processing step. The bottom-right panel shows where the observed Hβ line arises from – remarkably, Hβ in emission is produced close to the surface (e.g., due to collisional effects) and has little to do with the kinematics of the interior – this has critical implications for BH mass estimates based on Balmer lines.

We note that this physics (electron scattering as well as resonant Balmer scattering) is by no means exotic and is seen routinely in e.g., supernovae – see for instance Xu+92 for discussion of extreme Balmer decrements and resonant Balmer scattering in SN1987A occurring in a partially ionized medium like the one we propose here.

Another circumstantial way to see that resonance scattering of at minimum H-alpha (but likely also H-beta) must be happening is via the OI 8446 line in LRDs (e.g., Kokorev+25). This line occurs from an accidental resonance with Lyman-beta, and collisional excitation and recombination can be ruled out based on the absence of other OI lines. However, because Lyman-beta can fluoresce to H-alpha, if H-alpha is not optically thick, the OI fluorescence would not occur. Hence, H-alpha must be scattering.

Finally, a similar ionization structure as our proposed model has been simulated in detail recently by Chang+26. We have appended Fig. 15 below from this paper for convenience – please notice that electron scattering (predominantly in the green region) and resonant Balmer scattering (predominantly in the gray region) are occurring in the same simulated configuration.

[Figure Redacted]

-In the case of electron scattering, the claim made in this work does not appear to be novel. A recent study by Rusakov et al. (2026), published in Nature, applied their cocoon model to an ensemble of LRDs and demonstrated that electron scattering can account for the observed line broadening. The authors should clearly distinguish their results from this existing work or clarify what new insight their study provides beyond the findings of Rusakov et al.

Rusakov+26 have shown some LRDs to have exponential line profiles. This is interpreted as evidence for electron scattering *consistent* with a cocoon of dense, ionized gas. However, note that exponential line profiles are common among several classes of (un-cocooned) local AGN (Laor+06, see also discussion in Santos+25). Therefore, while the existence of electron scattering in ionized gas provides independent support for the proposed BH* picture, it is by itself not definitive.

The work at hand demonstrates ultra-deep Balmer breaks – which cannot be stellar – occur in nature. This discovery provides the critical empirical underpinning for a possible framework for why Little Red Dots are red. In particular, inspired by this source's remarkable properties we propose that optical light in LRDs may be powered by a similar family of BH*s that fade below the Balmer limit. Note that electron scattering proposed by Rusakov+26 is wavelength-independent and cannot account for the red color of LRDs, which in our picture is due to Balmer opacity of excited HI gas, not ionized gas as discussed in that work. The framework first outlined in this paper (LRD = Host Galaxy + Black Hole Star) has had remarkable impact on the literature and is now being routinely used to model the full LRD population as demonstrated in the following papers to name a few: e.g., Taylor+25, Barro+25, Golubchik+25, de Graaff+25b, Umeda+25, Yanagisawa+26, Sun+26, Wang+26.

-A similar concern was raised by Referee #1, who noted: "I find the extrapolation from this single source to the overall population of Little Red Dots rather extreme." Unfortunately, the authors have not made a substantive effort to address this issue. Rather than presenting observational evidence

for a broader population of LRDs, they rely primarily on analytical arguments and interpretations drawn from previous studies. Indeed, the authors themselves acknowledge that "the main value of the paper is the discovery of the object," rather than a demonstrated applicability of their conclusions to the LRD population as a whole. Given the lack of supporting evidence extending beyond this single source, the scope of the work remains limited. As such, while the discovery itself may be of interest, the manuscript does not provide sufficient justification for the broad claims required for publication in Nature. The study would be more appropriately placed in a specialized astronomy journal.

We agree that extrapolating from one source to the full population of LRDs is challenging and beyond the scope of this manuscript – however, it is still extremely valuable to propose a possible framework motivated by the properties displayed by this source. The community is finding this framework extremely impactful as evidenced by its widespread uptake (e.g., Taylor+25, Golubchik+25, de Graaff+25b, Umeda+25, Yanagisawa+26, Sun+26, Barro+26, Wang+26). In this revision we have sharpened the focus on the unique nature of this source and explicitly present the applicability to LRDs as an intriguing proposal to be followed up in subsequent studies. Please see our response to Referee #1 for more on this topic.

-The authors also appear to over-emphasize and over-interpret the BH phenomenon by associating it with quasi-stars. This assumption significantly constrains their interpretation of the source properties and may bias their conclusions. A more comprehensive and balanced analysis would have been possible had the authors seriously considered alternative scenarios already discussed in the literature—such as those presented by Rusakov et al. (2025, Nature), Pacucci et al. (2026), and related works—and compared their findings accordingly. Such a comparison would have provided a more robust understanding of the nature of the source. It is unclear why the authors are reluctant to engage with these alternative explanations and instead strongly advocate for their own hypothetical BH* model. Exotic objects such as quasi-stars have never been observationally confirmed and are increasingly regarded as disfavored from the standpoint of modern stellar evolution theory. As a result, interpretations relying heavily on such objects are inherently uncertain. This concern has already been raised by one of the referees, who noted: "I also want to comment that I do not find the expression of a black hole star very appropriate." Moreover, many of the quintessential stellar features highlighted by the authors can be naturally explained within the frameworks proposed in the aforementioned studies, without invoking quasi-stellar objects. Finally, it should be emphasized that the authors' entire interpretation is based on one-dimensional CLOUDY models, which fall short of capturing the complexity of realistic astrophysical environments. In particular, the absence of turbulence—known to have significant implications for line broadening and black hole mass estimates—raises further concerns about the robustness of the derived conclusions.*

The referee raises several interesting points that we address below. Broadly, we have noted in great detail the caveats associated with our simplistic modeling, and acknowledge the diversity of possible scenarios without favoring any one over the other at this early stage of discovery.

None of our conclusions and no part of this manuscript relies on the hypothetical "quasi-stars"/"quasi-stellar objects" (failed supernovae of supermassive stars that may or may not exist) as critiqued by the referee. Indeed, if invoking this specific scenario was our goal we would have called these objects quasistars in the first place instead of the general and empirical "black

hole stars". We list quasi-stars as only one among several possible channels to produce BH*s – e.g., we cite Alexander & Natarajan 2014's model placing a black hole within a gas-rich nuclear star as possibly producing BH*s as well as Liu+2025's model involving a super-Eddington flow enshrouding a black hole in addition to the quasi-star and quasi-stellar models (e.g., Santarelli+26, Begelman+26).

As discussed in the text, "BH*" as introduced in this work denotes the empirical phenomenon encompassing *all* channels that produce a spectrum that has features classically associated with black holes (e.g., broad Balmer lines) as well as features classically associated with stars including e.g., pseudo-thermal SEDs (this work, de Graaff+25b, Sun+26), Sun-like Balmer absorption series with absorption increasing down the series (this work, Torralba+25, d'Eugenio+25, Lin+25), and A-star-like Balmer breaks (this work, de Graaff+25a, Sun+26). Furthermore, there may be sources with even possible Cepheid-like stellar pulsations (Zhang+25, Cantiello+26), M-dwarf-like water absorption (Wang+26), and hints of a "Hayashi track" in the HR diagram (de Graaff+25b, Umeda+25, Kido+25, Liu+25).

The two scenarios highlighted by the referee also ultimately appeal to enshrouded BHs like the scenario presented in this work. In particular, Rusakov+26 argues for dense gas (at least along the line of sight) based on electron scattering, whereas Pacucci+26 (appearing on arXiv after the submission of our revised manuscript) uses a very similar CLOUDY modeling framework as in this work to produce Balmer break SEDs by appealing to a spherical shell of dense gas.

-I reiterate my earlier concern, as the authors still do not present any concrete evidence to support their claims about super-Eddington accretion. Which remains largely speculative and lack the quantitative justification required to substantiate this scenario. It is unclear on what basis the authors rule out the possibility of Eddington-limited accretion, which remains a viable and well-motivated alternative. Therefore, the conclusion in favor of super-Eddington accretion is not sufficiently supported.

We agree with the referee that super-Eddington accretion is extremely challenging to establish in a novel class of objects. The bolometric luminosity calibrations and black hole mass calibrations for BH*s are still being established – and indeed, we believe this work serves a useful purpose in drawing attention to this issue. We have carefully gone through the manuscript (e.g., in the abstract, Table 5) and ensured we do not give the readers the impression that super-Eddington accretion can be concluded, but is only one of various possibilities. Indeed, as already appreciated by Referee #2, we have acknowledged these limitations explicitly in the manuscript several times (e.g., we say *"Given the significant systematic uncertainties, these calculations must be seen as order of magnitude estimates to bracket the range of possibilities.").* We also now cite a variety of recent theoretical works endeavoring to explain BH*-like spectra (e.g., Liu+25) that find near-Eddington/Super-Eddington accretion to be a viable scenario for such sources.

-In conclusion, I am not convinced that the proposed BH scenario can be extended to a larger—let alone the entire—population of LRDs. Several of the key arguments, as discussed above, remain speculative and would require additional observational evidence to robustly establish the nature of LRDs. As it stands, the data presented in the manuscript do not justify such broad generalizations.*

We thank the referee for their comments that have strengthened this manuscript.

Referee #1 (Remarks to the Author):

I thank the authors for the improvements compared to the previous version and for focusing the paper more strongly on the source that has been detected, which I believe merits investigation and discussion in its own right, and I also appreciate that more details on how this source compares to the typically known AGN have been added. Nonetheless I also have some remaining concerns about the presentation, that I want to line out here:

1) I previously expressed my discomfort with the name black hole star. The authors have argued that this name is mostly a figure of epic and prosaic speech, which in part can be true. However, it is still important that this concept is clearly defined and applied in a consistent way throughout the manuscript, also to still have clarity on what we mean by it within the community. Within the manuscript, the authors essentially say that they model a black hole within an accretion disk of gas, which I believe very likely is the correct description of what they are doing. On the other hand, in the introductory bold-face paragraph, they define it as a "theoretical configuration proposed to rapidly grow black holes via super-Eddington accretion", refering then to references 10-14, which are references mostly refering to so-called quasi-stars or supermassive stars, which are very different from the disk-like configurations that would be more plausible. I believe this is at least misleading and could very easily misinterpreted, and the authors should really quote papers that really refer to disk-type structures and remove the references that refer to quasistars that correspond to a very specific hydrostatic equilibrium configuration which really has not been investigated here. Such configurations have been proposed for very early stages when the supermassive black hole was about to form, not as a general configuration for a black hole in an accretion disk, and to my knowledge there are not even theoretical studies that would suggest such a configuration to form within an accretion disk. I also believe, in case the possibility of a quasistar is really considered, that the type of modeling should be different, including at least a 1D model of the stellar structure of the quasi-star together with an atmospheric model that determines on what would be visible. If the authors have ideas on this specific case, perhaps they should rather explain these in another part of the manuscript or provide further details in the supplementary material, while the core summary should focus on what is clearly established.

All references to “Black Hole Star” have been removed from the manuscript.

2) My previous question on the properties of the object that formed here has been possibly misunderstood. My interest was about how to generate an object with such an extreme Balmer break as found here and why it appears to happen so rarely. Is the explanation only the gas column density from the authors point of view? What does it then mean if in this particular object, such a high gas column density is present? Is it only a rather peculiar initial condition? Or do we see a very short-lived temporary phase, that may have existed also in other sources, but not long enough to be observed? I understand that perhaps it cannot yet be answered fully, but i think nonetheless the question still should be raised.

We thank the referee for raising excellent questions about the origins of objects like MoM-BH*-1. It might not be that rare of a phenomenon, since the connection to Little Red Dots implies this may be a rather ubiquitous phenomenon. It is by chance, in this object that we are witnessing such a gas-enshrouded black hole in a low-luminosity galaxy – the remarkable contrast against the low-

luminosity of the host thus reveals the existence of this stage of BH growth. We agree that answering the questions of how these objects form in this first place is beyond the scope of the manuscript. While discussing various possibilities for the formation we have added the following sentence which is in the spirit of the referee's suggestion that the question still should be raised: "*For now, we note that the formation of such enshrouded BHs remains an open question.*"

3) I believe there is also something to be learned from the argument by the authors that we see a somewhat lucky circumstance that the black hole star is forming in a faint source. This certainly can be so, but the question is why is it faint. Is it that the stellar population is rather old, or are there simply less stars in that source? Does it mean that the mass and luminosity of the new black hole star is usually similar, irrespective of the properties of the host galaxy? That would be a possible useful point to make, if it is what the authors indeed intend to express.

We thank the referee for the thought provoking questions. The host galaxy does not appear to be an outlier in the M_{UV} vs. L_{O3} relationship, and may therefore simply be a generic <~0.1 L* star-forming galaxy that is being outshone. We do not mean to suggest that there is a universal BH* luminosity. There is a large span in black hole star properties ($L_{bol} \sim 10^{43-45}$ erg/s; de Graaff+25d, Sun+26, Lin+26) and MoM-BH*-1 ($L_{bol} \sim 10^{44.5}$) lies within the upper envelope of this distribution.

Referee #3 (Remarks to the Author):

-The authors state that "we agree that extrapolating from one source to the full population of LRDs is challenging and beyond the scope of this manuscript," yet a substantial portion of the Methods section remains devoted to comparisons with LRDs, particularly on pages 10–11 under the headings "SMBH Properties and Ramifications for LRD Parameters" and "Host Galaxy Properties." In fact, the terms "LRDs" and "Little Red Dots" appear nearly 40 times throughout the manuscript. This seems inconsistent with the statement in the referee response that "in this revision we have sharpened the focus on the unique nature of this source." The manuscript should be revised more carefully to emphasize the discovery of this source and its specific properties. Any discussion of possible implications for LRDs should be brief and clearly framed as speculative, given that the authors do not provide evidence supporting conclusions about the broader LRD population. Additionally, Figure 4 appears to be largely speculative and does not represent a central result of this study. Therefore, It should be removed it from the manuscript.

-Related to the previous point, the abstract also contains speculative statements that should be removed. For example: "If the BH merged with its brighter neighbor, it would resemble the recently discovered 'Little Red Dots'" and "SMBH masses of such sources may therefore be overestimated by orders of magnitude."*

We have sharpened the focus further and reduced the length of the manuscript, e.g., the discussion of resonant scattering in Little Red Dots vis-a-vis their Balmer decrements has been completely excised with the referee's suggestion in mind. Despite the speculative nature, for the reasons outlined in detail in our previous response we find Figure 4 (and the immediate implications of this exercise) to be an extremely useful direction that arises naturally from this source's properties and that is providing a powerful way of thinking about LRDs.

-The central premise of the BH-star scenario relies on the presence of a thick, dense gas envelope surrounding the black hole. The necessity of such an envelope appears to stem from attempts to reproduce the observed spectrum using Cloudy models. However, the authors do not explain how or under what physical conditions such an envelope would form, nor how long it could be sustained. The manuscript lacks a physical interpretation of this scenario, and relying on this assumption without adequate justification is not sufficient.

Please also see our response to Referee #1 on the topic of the origins of BH*s. The empirical discovery of this class of sources is the focus of our current manuscript (as encouraged also by the referee). Unraveling how exactly these sources form and what becomes of them is beyond the scope of this work and currently the subject of a large-scale community-wide effort.

- In their response, the authors state that the BH-star scenario is not based on quasi-stars, but more generally refers to a massive black hole enshrouded by dense gas, potentially including BHs in gas-rich stellar clusters, direct-collapse black holes forming in atomic cooling halos, or systems arising in the aftermath of galaxy mergers. However, after carefully reading the manuscript, I could not find a clear or consistent explanation of what is meant by the "BH-star" model in this broader context. The concept remains insufficiently defined. In addition, the literature review appears somewhat unbalanced. Most of the cited works relevant to this scenario focus on quasi-stars, which seems inconsistent with the authors' claim that their model is not based on quasi-stars. A more representative and clearly justified selection of references is needed.

-The new title, "A Black Hole Star" does not adequately reflect the primary result of the paper—namely, the discovery of a unique source exhibiting the largest Balmer break. I recommend that the authors revise the title to better highlight this original finding.

As noted above, the "BH-star" scenario is neither uniquely defined nor newly introduced in this work, despite the impression given by the manuscript. Rather, it appears to be one of several possible interpretations, as the authors themselves state: "BH as introduced in this work denotes the empirical phenomenon encompassing all channels." In this context, the use of the term "BH*" is misleading. The authors should drop this notation and instead adopt a more general and established description, such as a black hole enshrouded by dense gas.*

Please see our response to referee #1. No references to "black hole stars" exist in the updated manuscript.

-The authors continue to place strong emphasis on the super-Eddington scenario, as reflected in the abstract and throughout the manuscript, while giving little attention to the possibility of Eddington-limited accretion. This imbalance should be addressed more carefully to provide a more complete and balanced discussion of the viable scenarios.

In our updated Table 4 we report an Eddington-limited scenario as suggested by the referee. No calculation in the manuscript now assumes Super-Eddington accretion. The only place where such models are discussed are in comparisons with theory where gas-enshrouded scenarios occur.

-A major caveat in the treatment of resonant scattering is the absence of turbulence in the shell models used within the COLT radiative transfer framework. On the one hand, the authors invoke a turbulent velocity of ~500 km/s (see Table 4) to reproduce the observed spectrum; on the other hand, turbulence is not included in the COLT model. This apparent inconsistency must be addressed, as it has significant implications for the conclusions of the present work and cannot be deferred to future studies.

We have acknowledged this caveat in the text: “Strong turbulence is not accounted for in these toy models.” We have presented this model from the perspective of a first exploration of a novel object to give a sense for the broad physics that future work may then build on.
